# Slow lung clearance and limited translocation of four sizes of inhaled iridium nanoparticles

**DOI:** 10.1186/s12989-017-0185-5

**Published:** 2017-02-10

**Authors:** Alison Buckley, James Warren, Alan Hodgson, Tim Marczylo, Konstantin Ignatyev, Chang Guo, Rachel Smith

**Affiliations:** 1 Public Health England, Centre for Radiation, Chemical and Environmental Hazards, Harwell Science and Innovation Campus, Didcot, Oxfordshire, OX11 0RQ UK; 2 Diamond Light Source Ltd., Harwell Science and Innovation Campus, Didcot, Oxfordshire, OX11 0DE UK

**Keywords:** Nanoparticles, Inhalation exposure, In vivo study, Rat, Tissue distribution, Lung clearance, Toxicity

## Abstract

**Background:**

Concerns have been expressed that inhaled nanoparticles may behave differently to larger particles in terms of lung clearance and translocation, with potential implications for their toxicity. Studies undertaken to investigate this have typically involved limited post-exposure periods. There is a shortage of information on longer-term clearance and translocation patterns and their dependence on particle size, which this study aimed to address.

**Methods:**

Rats were exposed (<3 h) nose-only to aerosols of spark-generated radioactive iridium-192 nanoparticles of four sizes: 10 nm, 15 nm, 35 nm and 75 nm (count median diameter) (aerosol mass concentrations 17, 140, 430, and 690 μg/m^3^, respectively). The content of iridium-192 in the whole animal, organs, tissues, and excreta was measured at various times post-exposure to ≥ 1 month. Limited toxicological investigations were undertaken for the 10 nm aerosol using bronchoalveolar lavage fluid. Elemental maps of tissue samples were produced using laser ablation inductively coupled plasma mass spectrometry and synchrotron micro-focus x-ray fluorescence. The chemical speciation of the iridium was explored using synchrotron micro focus x-ray near-edge absorption spectroscopy.

**Results:**

Long-term lung retention half-times of several hundred days were found, which were not dependent on particle size. There was significant variation between individual animals. Analysis of bronchoalveolar lavage fluid for the 10 nm aerosol indicated a limited inflammatory response resolving within the first 7 days. Low levels of, particle size dependent, translocation to the kidney and liver were found (maximum 0.4% of the lung content). Any translocation to the brain was below the limits of detection (i.e. < 0.01% of the lung content). The kidney content increased to approximately 30 days and then remained broadly constant or decreased, whereas the content in the liver increased throughout the study. Laser ablation inductively coupled plasma mass spectrometry analysis indicated homogeneous iridium distribution in the liver and within the cortex in the kidney.

**Conclusions:**

Slow lung clearance and a pattern of temporally increasing concentrations in key secondary target organs has been demonstrated for inhaled iridium aerosol particles < 100 nm, which may have implications for long-term toxicity, especially in the context of chronic exposures.

**Electronic supplementary material:**

The online version of this article (doi:10.1186/s12989-017-0185-5) contains supplementary material, which is available to authorized users.

## Background

Rapid developments in the field of nanotechnology over the past decades, which have led to the production of an expanding range of nanomaterials being used in an increasing number of products, have been paralleled by growing concerns about the potential health effects of exposures to these materials. One such concern is that inhaled nanoparticles may behave differently to larger particles in terms of their clearance from the lung and translocation to secondary target organs and tissues, with concomitant implications for potential toxicity. This is a particular concern in relation to poorly soluble materials due to their potential biopersistence. A number of studies have investigated these issues and there appears a growing consensus that translocation is low, typically < 1% of lung deposition to key organs, for highly insoluble nanomaterials [1]. The majority of studies have, however, only considered limited post-exposure timescales. Furthermore, the situation in relation to lung clearance is unclear, with many studies indicating clearance timescales of the same order as larger particles (e.g. [2]) but a minority suggesting much slower clearance (e.g. [3, 4]). In both areas there is a lack of studies systematically investigating the effect of particle size. The majority of studies have been undertaken using micron-sized aerosols of agglomerated nanoparticles, which, whilst clearly relevant to some occupational exposure situations, are potentially less so for exposures of members of the public resulting from the use of products containing nanoparticles, as studies of consumer spray products have indicated the potential for exposures to nano-sized aerosols [5, 6]. In general there is a lack of information on long-term clearance and translocation patterns for such aerosols.

The potential toxic effects of inhaled nanoparticles on the lung and other organs and tissues depend on a number of factors, including: amount (dose); location (e.g. sub-organ/tissue structure, cell type, subcellular); and chemical state, in particular whether the material remains in its original nano-particulate form or is in an alternative form(s) as a result of dissolution and subsequent chemical reactions in vivo. Information on these factors is therefore an important input to an assessment of toxic potential.

The primary objective of this study was to investigate the effect of aerosol particle size on the long-term clearance and translocation of nanoparticles following inhalation. A secondary aim was to explore the localisation and chemical state of the inhaled material. Rats were exposed in a nose-only inhalation system to radioactive iridium-192 aerosols of four sizes, chosen to cover the majority of the nano-size range (nominal sizes: 10, 15, 35, and 75 nm), and the content of iridium-192 in the whole animal, a number of organs and tissues, and excreta was measured at various times post-exposure. Toxicological investigations were also undertaken for the 10 nm aerosol using bronchoalveolar lavage fluid (BALF). A number of techniques were used to investigate the localisation and chemical speciation of the inhaled iridium, using samples from the iridium-192 inhalation experiments and additional inhalation experiments using non-radioactive iridium aerosols (10 nm and 75 nm). Tissue samples were imaged using transmission electron microscopy and elemental maps produced using both laser ablation inductively coupled plasma mass spectrometry (LA-ICP-MS) and synchrotron micro focus x-ray fluorescence (μ-XRF). In addition, synchrotron micro focus X-ray near-edge absorption spectroscopy (μ-XANES) was used to explore iridium speciation.

Iridium nanoparticles are currently found in only a limited number of consumer products, although potential uses being investigated, including catalysts (e.g. [7]), could lead to more widespread exposures. However, the objective of this study was not to investigate iridium per se but, as has been the case in other studies [8], iridium nanoparticles were chosen as a model poorly soluble particle (in bulk it is one of the most corrosion resistant and insoluble elements), with the aim that the study conclusions would be generally relevant to other poorly soluble nanoparticles.

## Results

### Aerosol characterisation

**Fig. 1:**
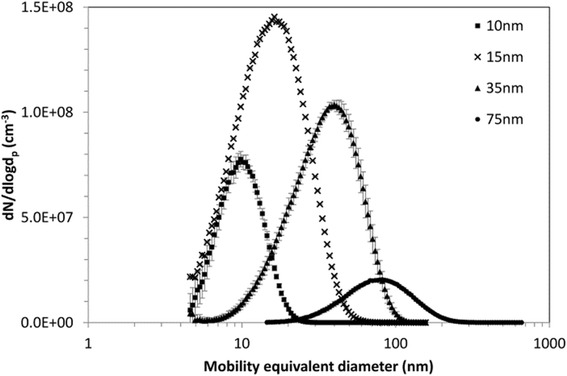
Average mobility equivalent size distributions of iridium-192 aerosols. Error bars shown are the standard deviation

The characteristics of the iridium-192 aerosols for the main study are given in Table 1 with average particle size distributions in Fig. 1. Representative transmission electron microscopy (TEM) images of aerosol particles collected during exposures show that the spark generated aerosols consist of chain agglomerates (Additional file 1: Figure S1). Primary particle size was consistent across all agglomerate sizes investigated with diameters in the range 1.2 nm to 2.9 nm. The 15 nm iridium-192 aerosol used for the supplementary study was similar to that used for the main study, with a count median diameter (CMD) of 16.6 ± 0.1 nm (Additional file 1: Table S1). The characteristics of the non-radioactive iridium aerosols were also similar to those used in the main study (Additional file 1: Table S2).

**Table 1:** Aerosol characteristics for main iridium-192 study

Nominal particle diameter (nm)	CMD (nm)	GSD	Particle number concentration (x 10^13^ #/m^3^)	Activity concentration (kBq/m^3^)	Mass concentration (mg/m^3^)
10	9.8 ± 0.1	1.37 ± 0.01	2.85 ± 0.10	490 ± 90	0.17 ± 0.03
15	15.6 ± 0.3	1.67 ± 0.01	9.09 ± 0.14	2380 ± 250	1.39 ± 0.15
35	33.4 ± 2.1	1.69 ± 0.02	5.63 ± 0.13	590 ± 140	4.28 ± 0.84
75	76.3 ± 1.9	1.62 ± 0.01	1.10 ± 0.03	600 ± 100	6.92 ± 1.38

Aerosol particle size distribution expressed as count median diameter (CMD) and geometric standard deviation (GSD). Values presented as mean ± standard deviation. Concentrations for the 10 nm aerosol have been corrected for system losses (see Methods for details)

### Radionuclide content of biological samples

The iridium-192 content of organs, tissues, blood samples and excreta at various times post-exposure are contained in Additional file 2. Results for a number of organs and tissues have been used to derive size-dependent deposition efficiencies in the respiratory tract and its component parts (head airways, total lung, tracheobronchial region and alveolar region) and these have been reported previously [9].

#### Lung clearance

The iridium-192 content of the lung, gastrointestinal tract (GIT) and pelt and also the total in and on the rat measured during the first two weeks post-exposure (Additional file 1: Figure S2), indicates that initially for the 10 nm aerosol the iridium-192 in the lung is similar to that in the GIT and pelt, however, for both the 15 nm and 35 nm aerosols the pelt and GIT contents are similar but approximately a factor of between 2 and 5 times lower than the lung content. For the 75 nm aerosol at the first time point the lung content is also approximately double that of the pelt and GIT, which are similar (Additional file 2). The pelt and GIT content reduce significantly during this time period so that by the end of the first few days the lung content accounts for more than 97% of the total.

**Fig. 2:**
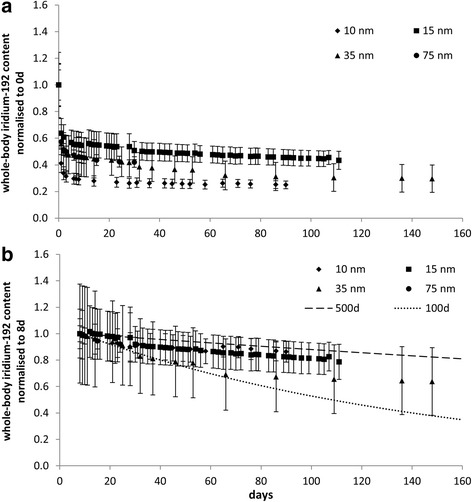
Average whole-body iridium-192 content for 10 nm, 15 nm, 35 nm, and 75 nm aerosols: (**a**) normalised to whole body content at 0 h and (**b**) normalised to whole body content at 8 d, with theoretical plots for retention half-times of 100 days (dotted line) and 500 days (*dashed line*)

**Fig. 3:**
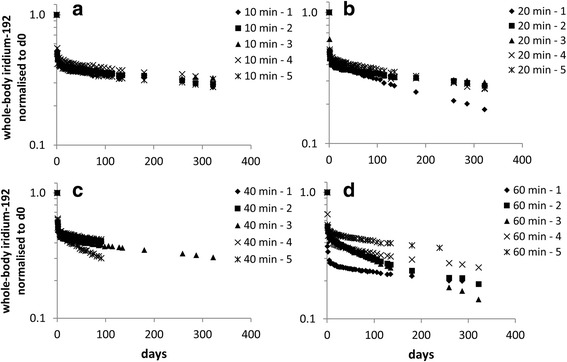
Whole-body iridium-192 content of individual animals normalised to whole-body content at 0 h for: (**a**) 10 min, (**b**) 20 min, (**c**) 40 min, and (**d**) 60 min exposures to 15 nm iridium-192 aerosol

The whole-body iridium-192 content for all particle sizes shows an initial fast clearance phase during the first few days, reflecting both clearance from the pelt and from the head and ciliated airways, followed by a much slower clearance phase with a half-time of between approximately 100 and 500 days (Fig. 2). The iridium-192 content of the lung measured at various times post-exposure (Additional file 1: Figure S3) also indicates slow clearance from the lung suggesting half-times in excess of 100 days for all particle sizes. The average whole-body iridium-192 content from the supplementary study with the 15 nm aerosol for four different exposure durations (and thus deposited doses) shows a similar pattern of slow clearance for all four groups, consistent with that seen in the main study (Additional file 1: Figure S4). Interestingly, the results for individual animals (Fig. 3) indicate an animal specific variation in clearance patterns which becomes more pronounced with increasing exposure duration.

#### Bronchoalveolar lavage fluid

Bronchoalveolar lavage was performed on animals exposed to the 10 nm (days 0, 3, 7, 30 and 90 post-exposure) and 35 nm (days 3 and 28 post-exposure) aerosols. The fraction of the iridium-192 in the lung removed by lavage showed little variation with time following exposure (average 35%) - at day 0 the majority was associated with the supernatant, however, at later times the majority (>80%) was associated with the cellular fraction (Additional file 1: Table S3).

#### Excretion

The excretion of iridium-192 (Additional file 1: Figure S5) is predominantly via faeces, with a rapid reduction within the first week consistent with an initial fast clearance from the head airways, ciliated airways and pelt (via grooming). Urinary excretion levels are lower and show a less significant reduction with time. Expressed as a function of the initial lung deposit, levels are similar for the 10 and 15 nm aerosols but significantly lower for the larger aerosol particles.

#### Translocation

**Fig. 4:**
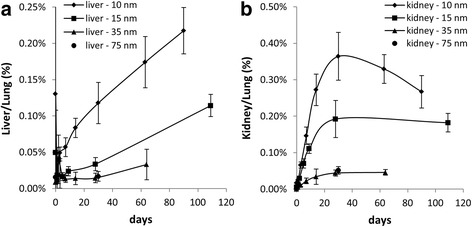
Iridium-192 content of (**a**) liver and (**b**) kidney normalised to contemporaneous lung content for 10 nm, 15 nm, 35 nm, and 75 nm iridium-192 aerosols

The iridium-192 in the liver and kidneys as a function of the lung content are presented in Fig. 4. The absolute levels of translocation are low (<1%). For kidney the peak is approximately 0.35% and for liver 0.20%. For both organs translocation is higher for smaller particle sizes, with the results for 35 nm and 75 nm aerosols very similar. The temporal trend for liver is a constant increase over the time period investigated (max 110 days), whereas that for kidney indicates a peak at around 30 days followed by a levelling off or reduction. The iridium-192 levels in the brain were < minimum detectable activity (MDA) for all particle sizes. Iridium-192 in spleen and blood samples was < MDA for the two larger particle sizes; for the others some samples were > MDA but with no clear pattern. The iridium-192 content of the carcass was typically 0.1 to 0.6% of the lung level, with no clear pattern with respect to particle size or time post-exposure.

### Cytotoxicity

Analysis of BALF from animals exposed to the 10 nm aerosol and sacrificed at days 3 and 7 post-exposure indicated limited toxicity. There was a small increase in the neutrophil population at day 3 (2.4 ± 0.7%) compared with control group, which recovered by day 7 (Additional file 1: Figure S6). There was no significant change in total cell numbers (4.1 ± 0.4 × 10^6^) or numbers of macrophages or lymphocytes. Only at day 0 was there a significant increase in lactate dehydrogenase (LDH) which recovered by day 3 (Additional file 1: Figure S6).

### Electron microscopy

Electron microscopy images and associated elemental analysis of BALF cell samples from the 75 nm aerosol exposed animals at 1, 5 and 30 d post-exposure clearly indicate the presence of iridium nanoparticles within BALF cells (Additional file 1: Figures S7, S8). Dense nano-sized particles of similar morphology to the aerosolised particles were also seen in other samples (particle sizes 10 nm (lung and BALF cells) and 75 nm (lung); 1, 5, and 30 d post-exposure (data not shown)), however, it was not possible to confirm that these were iridium as elemental analysis of particles in samples was not undertaken for these samples.

### Laser ablation ICP-MS

**Fig. 5:**
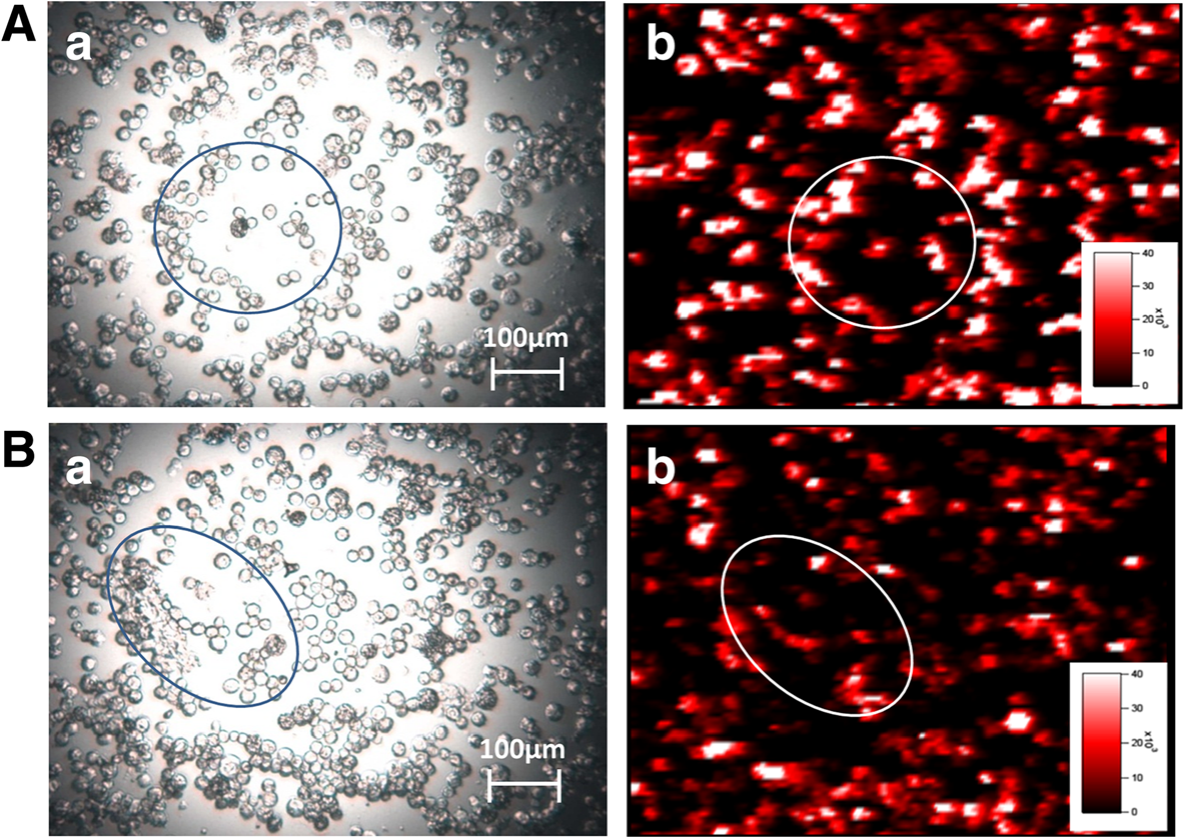
Laser ablation inductively coupled plasma mass spectrometry (LA-ICM-PS) generated elemental maps of BALF cell samples from rats at 3 days post-exposure to 10 nm iridium aerosol (A) and 589 days post-exposure to 35 nm iridium-192 aerosol (B); showing light microscopy image of the cell sample analysed (a) and the distribution of iridium (b). Scales are in counts per second. Some areas have been highlighted to aid comparison

**Fig. 6:**
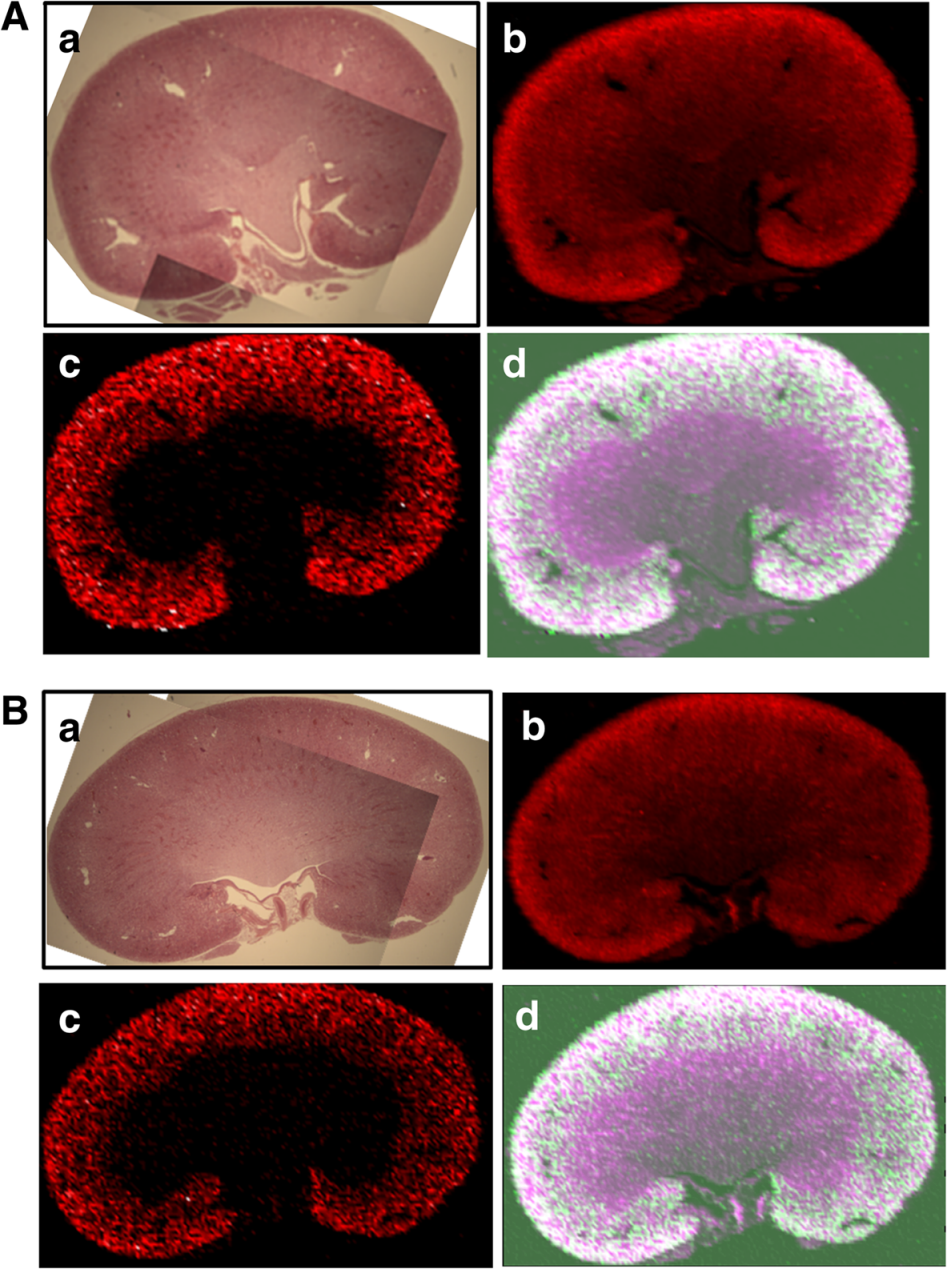
Laser ablation inductively coupled plasma mass spectrometry (LA-ICM-PS) generated elemental maps of kidney samples at 30 days post-exposure from rats exposed to 10 nm (A) and 75 nm (B) iridium aerosols; showing light microscopy image of the kidney section analysed (a) and the distribution of copper (a), iridium (b) and an overlay of the two plots (c) (copper, *magenta*; iridium, *green*; both, *white*) Scales are in counts per second. The copper distribution map is included to enhance visualisation of the localisation of the iridium; results from control animals (not shown) indicate no iridium is present and the same overall pattern of copper distribution

Elemental maps of iridium (and other elements for comparison purposes) were produced for sections of lung, liver, kidney, and brain, and BALF cells. Iridium is clearly seen in the lung at 1, 5 and 30 days post-exposure for both the 10 nm and 75 nm aerosol (Additional file 1: Figures S9, S10). Elemental maps of BALF cells samples for the 10 nm aerosol at 3 days post-exposure and 35 nm aerosol at 589 days post-exposure show similar patterns of iridium association with BALF cells (Fig. 5). At 30 days post-exposure iridium is clearly seen in the kidney (Fig. 6) and liver (Additional file 1: Figure S11) for both the 10 nm and 75 nm aerosols. The iridium is concentrated in the kidney cortex. Further investigation of a small area of cortex using a reduced spot size failed to establish whether the iridium was associated with particular structures. The results for the liver indicate a homogenous spread, however, at this resolution it is not possible to identify whether the iridium is associated with specific organ structures.

### Synchrotron X-ray spectroscopy

**Fig. 7:**
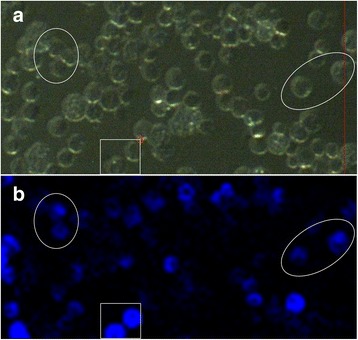
Elemental μ-XRF map of BALF cell sample from animal exposed to 35 nm iridium aerosol at 589 d post-exposure, obtained using the I18 beamline at the Diamond Light Source (pixel size 4 μm × 4 μm); showing light microscopy image of the BALF cell sample analysed (**a**) and the distribution of iridium (*blue*) (**b**). Some areas have been highlighted to aid comparison

**Fig. 8:**
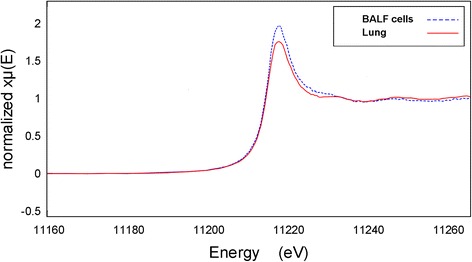
Iridium L(III)-edge μ-XANES spectra derived from iridium-rich pixels from samples of BALF cells shown in Fig. 7 and lung tissue at 1 day post-exposure to 10 nm iridium aerosol. The lung spectrum is the sum over 6 pixels and that for the BALF over 10 pixels

Elemental maps (μ-XRF) were produced for one lung section (10 nm, 1 day post-exposure (Additional file 1: Figure S12)) and two BALF cell samples (10 nm, 3 days post exposure (Additional file 1: Figure S12) and 35 nm, 589 days post-exposure (Fig. 7)), which identified areas containing iridium from which μ-XANES spectra were collected. The μ-XANES spectra are difficult to interpret. Those for the lung section and the 589 day BALF cell sample are very similar (Fig. 8), which could suggest limited change in particle state following exposure, however, those for the standards are also very similar (Additional file 1: Figure S13). Additional studies are required to produce: (a) additional sample spectra, and (b) spectra from standards of compatible form, such that detailed fitting programs can be used to compare sample spectra to standards.

## Discussion

This study investigated the effect of inhaled aerosol particle size (range 10–75 nm) on lung clearance and translocation to other organs and tissues.

### Relevance of aerosol mass concentrations

The aerosol mass concentrations used in this study (range 0.2 to 6.9 mg/m^3^) are typically higher than those suggested by limited measurements of consumer spray products (e.g. 17 μg/m^3^ nano-fraction [5]). The masses deposited in the lung (in the μg range) are two orders of magnitude greater than available estimates of deposited doses per product use [5, 10], and thus broadly consistent with the mass deposited in the lung by daily use of a product for 3 or 4 months. However, it must be recognised that the actual lung burden after this time would differ as a result of clearance over this period.

### Lung clearance

Assessment of the content of organs and tissues indicated that after the first few days post-exposure the majority of the iridium-192 (>97%) was retained in the lung, thus measurements of the whole-body content were representative of those in the lung. These show slow long-term clearance of iridium from the rat lung (i.e. retention half-times of several hundred days) with no dependence on particle size within the range considered (Fig. 2). For the 15 nm aerosol there was no dose dependent effect on clearance over the range of deposited dose used (0.5–3.2 μg/animal – Additional file 1: Table S4). The variation in clearance rates between individual animals exposed to the 15 nm aerosol for 10, 20, 40 and 60 mins, tended to increase with exposure duration (approximate range 200–500 days retention half-time) (Fig. 3).The results may reflect the effect of changes in factors such as animal position, behaviour and breathing patterns with time and/or inherent variability within the population.

Reported lung retention half-times following the inhalation of low concentrations of low solubility nanoparticles (e.g. TiO_2_, Carbon Black, CeO_2_) in rats are typically in the range 40–60 days (e.g. [11–13]), i.e. comparable to those for larger particles and significantly shorter than found in this study. Slower clearance rates have been reported for such materials but typically only at high aerosol concentrations and have been interpreted as indicating ‘lung overload’ associated depression of alveolar macrophage clearance [11–13]. The majority of such studies have, however, been undertaken using aerosols with agglomerated particles in the micron-size range.

There are few studies which have directly addressed the clearance of inhaled nano-sized aerosol particles. Two studies using silver nanoparticle aerosols (15–17 nm) indicated rapid clearance [14, 15] and studies using a gold nanoparticle aerosol (16 nm) and a C_60_ (fullerene) aerosol (55 nm) estimated lung retention half-times of, respectively, 30 days [16] and 26 days [17]. In all these studies, however, clearance was only considered to 7 days post-exposure, so there is a measure of uncertainty surrounding the extrapolation of these estimates to long-term clearance, especially given the dominance of fast mucociliary clearance during the initial days post-exposure. One study investigated the clearance of gold nanoparticles to 28 days post-exposure [4]. Male Sprague-Dawley rats were exposed in a nose-only system to aerosols produced by atomizing 1% citrate dispersions containing small (13 nm) or large (105 nm) gold particles. The aerosol particles produced were predominantly in the nano-size range for both materials, however, the size of the aerosolised gold particles is unclear as the system produced a large number of citrate aerosol particles, which makes interpretation of the particle size distribution data difficult. The study estimated a retention half-time of 180 days for the larger primary particles, which is comparable with that found in this study, although with only 3 data points there is clearly significant uncertainty surrounding this estimate. A retention half-time for the smaller particles of 44 days was also estimated, although there is significant uncertainty surrounding this as the concentration of gold in the lung for the 3 d and 28 d groups was higher than for the 1 d group.

One of the few inhalation studies to investigate longer-term clearance of nano-sized aerosols also used iridium-192 particles produced by spark generation [2]. Aerosols of two sizes, 15 nm and 80 nm, were delivered by endotracheal tube to male Wistar rats for between 1 and 1.5 h (reported lung burdens 2 to 4 μg/rat [8]). The whole-body and various organ iridium-192 contents were measured at intervals up to 6 months post-exposure. Using the whole-body measurements from day 3 post-exposure a retention half-time of approximately 100 days was estimated, broadly in line with clearance for larger particle sizes and markedly lower than that seen in this study. It is not immediately clear why two very similar studies should produce different clearance rates. The sex of the animals used was different, but whilst there has been some suggestion of a sex difference in translocation [18, 19] there is no indication of such for lung clearance. The main difference between the studies is the exposure route. Endotracheal intubation results in the delivery of the whole aerosol directly to the lung, by comparison, initial delivery to the nose means that the aerosol ‘seen’ by the lung is modified as a result of deposition within the nasal cavities with fewer of the smaller particles present (45% of particles < 6 nm - Additional file 1: Figure S14). This could modify initial clearance rates, as there are indications that particles < 6 nm can enter the blood stream quickly [20], but it is unclear how this could affect long-term clearance rates. The results of the two studies also differed with respect to the lavageable fraction: in our study approximately 30 to 40% of the iridium-192 within the lung could be removed by bronchoalveolar lavage at all time points considered, whereas in the earlier study the lavageable fraction (46% at 0 h) decreased markedly during the first 6 h to 19% and further to 6% at 21 days and thereafter (to 6 months) remained fairly constant at this level [21].

### Translocation and excretion

The results of this study indicate low levels of translocation of iridium from the lung to the kidney and liver (maximum 0.4% of lung burden) with translocation to kidney higher than liver. Translocation decreased as particle size increased but was similar for the two larger sizes. The temporal trend for liver is a constant increase, whereas that for kidney indicates a peak at around 30 days with a levelling off or reduction after this time. Levels of activity in the brain were below the limits of detection (i.e. < 0.01% of the activity in the lung) and patterns for blood and spleen were unclear.

These low levels of translocation are consistent with the results of a recent review which concluded that nanoparticle translocation to secondary target organs following inhalation is typically < 1% of lung deposition for insoluble nanomaterials [1]. However, the majority of the studies reviewed only considered limited post-exposure timescales and used micron-sized agglomerated aerosol particles. There are very few studies of the translocation of inhaled nano-sized aerosol particles and the results, although confirming overall low translocation levels, show significant variation. For example, one study using an aerosol of gold nanoparticles (<5 nm) and Sprague-Dawley rats found translocation only to the kidney with higher values for female than male [19], whereas a further study from the same group using aerosols produced by atomizing 1% citrate suspensions containing small (13 nm) or large (105 nm) gold nanoparticles found gold levels above control for the lung, spleen, liver and brain for the small aerosol only at day 1, with only the lung and spleen levels significant at day 28; there was no significant increase in kidney levels [4]. Another study exposed male Wistar rats to aerosols of gold nanoparticles of two primary particle sizes, 7 nm and 20 nm, but with similar aerosol particle agglomerate sizes, respectively 46 and 42 nm [22]. The results for kidneys and liver indicated translocation in the range 0.1–0.4%, similar to that found in this study; however, they also found translocation to a wide range of other organs and tissues including parts of the brain. A study using iridium-192 nanoparticle aerosols found translocation to secondary organs was lower for the 80 nm aerosol than the 15 nm aerosol [8], which is consistent with the size dependent pattern found in this study, however, the degree of translocation was higher at 7 days by approximately an order of magnitude than that found in this study. The temporal pattern, i.e. low levels of translocation to liver, spleen, brain and kidneys at 3 weeks, reducing thereafter [2], also differs from that found in the current study. As indicated above, it is not clear why such similar studies would produce different results. The increased percentage of smaller particles present for the endotracheal delivery may have increased the initial translocation, but, as indicated earlier, the difference in mass terms is small. The use of animals of a different sex may have had an influence, a number of studies have indicated sex differences in translocation to kidneys [18, 19], but neither of these factors can fully explain the differences.

There are few studies that have investigated excretion following the inhalation of nano-sized aerosols. We found iridium excretion is predominantly via faeces, consistent with other studies [2, 22], for which there was no evidence of a size dependent effect. In line with the translocation results, there was a suggestion of a size-dependent effect for urinary excretion, with this being greater for the two smaller particle sizes. A study to investigate the renal filtration threshold for metal based nanoparticles used quantum dots of 6 sizes (hydrodynamic diameter 4.4 to 8.7 nm) administered intraveneously and found that only particles < 5.5 nm were excreted [23]. The aerosol particle size distributions all have some fraction < 5.5 nm, with the proportion decreasing as the average particle size increases. Thus the results may reflect the excretion of this small particle fraction, however, the iridium in urine may have been in ionic rather than particulate form.

### Localisation and speciation

A secondary objective of this series of experiments was to explore the use of a number of techniques to explore localisation and chemical speciation. TEM identified iridium particles within BALF cells and lung tissue. LA-ICP-MS analysis of lung and BALF cell samples clearly indicated the presence of iridium. At low resolution the distribution of iridium was homogenously distributed throughout the lung. At higher resolution the iridium is clearly seen at short times post-exposure associated with the epithelial surface. BALF cell samples indicate the presence of iridium in cells, at short and long times post-exposure (Fig. 5). The μ-XRF elemental maps of lung tissue and BALF cells showed a similar pattern. The μ-XANES analysis of μ-XRF iridium ‘hot spots’ in a lung sample at 1 day post-exposure and a BALF sample at 598 days post-exposure show little change in the chemical speciation suggesting limited if any dissolution. However, systematic analysis of further samples is necessary to confirm these findings. The LA-ICP-MS analysis indicates the presence of iridium in the liver and kidney (Fig. 6), no iridium was seen in the brain (results not shown). For liver the iridium was homogeneously distributed, whilst that for the kidney showed concentration in the cortex. The intention is to further investigate the use of μ-XANES to explore speciation in these tissues.

### Implications for toxicity

Limited toxicological investigations (10 nm aerosol only) were carried out as part of this study. These indicated that inflammation within the lung shortly after exposure was low, resolving within a week, consistent with findings from a similar study using iridium-192 aerosols [21]. However, slow clearance from the lung suggests the potential for accumulation within the lung following chronic exposure, which requires further investigation. Although the quantities of material translocated to the liver and kidney are small, if this pattern were repeated for long-term exposures then levels could increase significantly, and this possibility also requires further investigation. The potential toxicological implications of any long-term build-up of concentrations within the lung and other organs are unclear and would require further investigation. The findings of a recent review [24] suggest that there is currently no convincing evidence for systemic toxicity following exposure to low solubility nanomaterials, however, they indicate that it is currently an open question whether long-term low dose accumulation could lead to material levels high enough to produce any systemic effects.

## Conclusions

This study found long-term lung retention half-times for iridium of several hundred days following exposure to nano-sized aerosols of iridium, which were not dependent on particle size in the range considered (10 nm to 75 nm). Analysis of BALF for the 10 nm aerosol indicated a limited inflammatory response resolving within the first 7 days. Low levels of, particle size dependent, translocation (maximum 0.4% of the lung burden) of iridium to the kidney and liver were found. Any translocation to the brain was below the limits of detection (i.e. < 0.01% of lung burden). The kidney content increased to approximately 30 days and then remained broadly constant or decreased, whereas the content in the liver increased throughout the study. LA-ICP-MS analysis indicated homogeneous iridium distribution in the liver and within the cortex in the kidney.

If the pattern of slow lung clearance and temporally increasing concentrations in key secondary target organs were repeated for long-term exposures then levels could increase significantly, and this possibility requires further investigation. The potential toxicological implications of any such accumulation would also need investigation. Understanding the clearance and translocation processes for the inhaled iridium nanoparticles requires more information on the localisation and chemical speciation of the iridium, in particular to understand whether particle dissolution is significant – to help address, for example, possible hypotheses such as that slow lung clearance is a result of dissolution followed by iridium binding to specific proteins. Initial investigations reported here have provided some relevant information but further studies are required.

Iridium was chosen for this study as a model poorly soluble material, as in bulk it is one of the most corrosion resistant and insoluble elements. However, the clearance results obtained differ from those for some other poorly soluble nanomaterials (albeit typically obtained using micron sized agglomerate aerosols), therefore caution must be exercised in extrapolating from these results for iridium to other materials. Additional studies using other materials are required to further explore the biokinetics of inhaled nano-sized aerosols of poorly soluble materials.

## Methods

### Experimental design

Details of the experimental plan for the main and supplementary studies are presented in Table 2. For the main study the intention was for the exposure duration to be the same (1 h) for all aerosols, however, the requirement to deliver a measureable quantity of radionuclide, and limitations imposed due to radioactive decay of the iridium-192 electrodes resulted in longer exposure durations for most of the groups.

**Table 2:** Experimental design

Nominal particle diameter (nm)	Exposure duration (mins)	Total animals	Post-exposure sacrifice time (group size)
*Main study – Clearance and translocation*			
10*	120	36	0 h (6), 24 h (4), 3d (4), 7d (4), 14d (4), 30d (5), 63d (4), 90d (5)
15	75	35	0 h (10), 24 h (5), 2d (4), 5d (4), 9d (4), 28d (4), 109d (4)
35	180	36	0 h (6), 24 h (6), 3d (4), 7d (4), 14d (4), 28d (4), 64d (4), 589d (4)
75	60	9	0 h (5), 30d (4)
*Supplementary study A – Clearance*			
15	10	5	
15	20	5	
15	40	5	
15	60	5	
*Supplementary study B – Additional samples for imaging*			
10	120	6	24 h (2), 5d (2), 30d (2)
75	60	6	24 h (2), 5d (2), 30d (2)

*For the 10 nm study additional animals (system controls) were exposed in the same system to gases without nanoparticles for 120 mins and sacrificed at 3 and 7 days post-exposure

### Nanoparticle aerosol production and exposure system

**Fig. 9:**
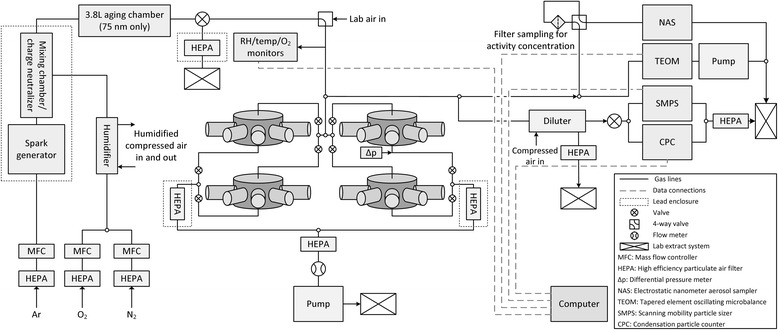
Schematic of exposure system (note that aging chamber was used for 75 nm aerosols only, for non-radioactive iridium aerosols the SMPS and NAS sampled directly from a chamber port, and TEOM^TM^ malfunctioned for 15 nm aerosol)

A schematic diagram of the nose-only inhalation exposure system is shown in Fig. 9. Aerosols of nano-sized iridium particles were generated using a spark generator (DNP 4000, Palas, Karlsruhe, Germany) with iridium wire electrodes held in custom made electrode holders. The sparking frequency used was between 90 Hz and 300 Hz and, if necessary, was adjusted during exposures to maiantain a constant aerosol size. Argon (Zero Grade Argon 99.999% minimum purity, BOC Industrial Gases, Guildford, UK) was filtered and delivered to the spark generator at a constant flow rate controlled by an argon calibrated mass flow controller (MKS, 1179A Mass-Flo®, Andover, MA, USA). The aerosol from the generator passed into a stainless steel neutralising and mixing chamber, containing a krypton-85 source (NER8180 capsule, 740 MBq (26/10/2009), Eckert & Ziegler Isotope Products GmbH, Berlin, Germany), where it was mixed with oxygen and nitrogen. Oxygen and nitrogen (99.999% minimum purity, BOC Industrial Gases, Guildford, UK) were filtered and the flow controlled using calibrated mass flow controllers (MKS, 1179A Mass-Flo®, Andover, MA, USA). Before mixing the oxygen and nitrogen were humidified using a Nafion® gas dryer (Perma Pure PD-Series™, Toms River, NJ, USA) to achieve a relative humidity after mixing with the aerosol of at least 30%. To achieve the largest agglomerate particle size additional aerosol aging was required and a 3.8 L stainless steel aging chamber, with an aerosol residence time of about 45 s, was introduced after the neutralising and mixing chamber.

The resulting aerosol entered a custom built nose-only exposure manifold (EMMS, Bordon, UK) [9]. This comprises four nose-only exposure chambers, each with 9 ports for animals or sampling probes. Depending on the number of animals to be exposed, between 1 and 4 chambers can be used, with those not in use isolated from the system. The rats are held in restraining tubes attached to the ports. The aerosol exhaust is filtered and the exposure manifold is inside a glove box held at a negative pressure of −3 in. H_2_O to provide secondary containment of the aerosol. Flow rates to the manifold were set to ensure delivery of at least 2 to 3 times the respiratory minute volume of the exposed animals (i.e. at least 0.5 L/min per exposure port) [25]. The oxygen concentration, temperature and relative humidity of the gas delivered to the exposure chamber were continuously measured during exposures using a MX300 medical oxygen analyser (Teledyne Analytical Instruments, City of Industry, CA, USA) and a HMT333 temperature/relative humidity sensor (Vaisala, Boulder, CA, USA). Across all experiments, the delivered gas temperature, relative humidity and oxygen concentration ranged between 19.0 and 23.5 °C, 30–55%, and 21.0–21.5%, respectively.

### Nanoparticle aerosol characterisation

For the main experiments using radioactive iridium, aerosol activity concentrations were determined using Pallflex® emfab™ filters (Pall Life Sciences, Ann Arbor, MI, USA) held in a 47 mm anodized aluminium filter holder (Pall Life Sciences, Ann Arbor, MI, USA). The aerosol was drawn through the filter at between 0.2 and 1.2 Lmin^−1^ for between 30 s and 2 mins, depending upon the electrode specific activity and aerosol mass concentration. Mass concentrations were derived from the activity concentrations using the specific activity of the electrodes. For the experiments using non-radioactive iridium electrodes, the average aerosol mass concentration was determined gravimetrically using the same filter sampling method but the aerosol was drawn through the filter for the duration of the experiments at sampling flowrates of 1 Lmin^−1^ and 0.25 Lmin^−1^ for the 10 nm and 75 nm experiments respectively. A TEOM™ ambient particulate monitor (Model 1400a, Thermo Scientific, Franklin, MA, USA) was used to continuously monitor the aerosol mass concentration delivered to the exposure manifold at a sampling flowrate between 0.5 and 1 Lmin^−1^ for all aerosols except the 15 nm, when it malfunctioned. The number concentration and particle size distribution of the aerosol delivered to the exposure manifold were also continuously measured during exposures using a condensation particle counter (CPC model 3775, TSI Inc., Shoreview, MN, USA) and a scanning mobility particle sizer (SMPS model 3936 N76, TSI Inc., Shoreview, MN, USA) with a differential mobility analyser (DMA) (model 3081, TSI Inc., Shoreview, MN, USA) for the 75 nm aerosol and a nano-DMA (N-DMA model 3085, TSI Inc., Shoreview, MN, USA) for the others. The morphology of the aerosol particles delivered to the manifold was determined with high resolution transmission electron microscopy (TEM) (JEOL 3000 F, JEOL Inc., Tokyo, Japan). Samples for TEM were taken directly onto 400 mesh copper TEM grids with lacey carbon film using an electrostatic precipitator (TSI 3089 nanometer aerosol sampler (NAS), TSI Inc., Shoreview, MN, USA). Sampling took place throughout the exposure for the 10 nm and 15 nm aerosols and at a number of times during the 35 nm and 75 nm exposures for a few minutes on each occasion.

Using the standard experimental configuration described above, the aerosol delivered to the manifold was continuously monitored during exposures; however it was not possible to monitor the aerosol delivered to the exposure chamber ports (point of administration (POA)) during exposures. Experimental analyses undertaken to determine the link between the aerosol delivered to the manifold and that delivered to each port (POA) indicated that for all but the smallest aerosol particle size the concentrations were approximately the same, but for the smallest aerosol particle size (10 nm) losses occurred resulting in the requirement for a correction factor of 0.72 ± 0.04 [9]. For the experiments using non-radioactive iridium aerosols the system was modified to allow particle size distribution measurements and sampling for TEM to be carried out directly from a chamber port, otherwise characterisation of the aerosol was undertaken as indicated above.

### Iridium electrodes

The iridium electrodes comprised 5 mm lengths of 0.8 mm diameter iridium wire (99.9% purity, Goodfellow Cambridge Ltd, Huntingdon, UK). To produce radioactive electrodes these were neutron activated at the Imperial College Reactor Centre, Ascot, UK. The primary radionuclide produced is iridium-192, which has a half-life of 74 days and decays principally by beta emission producing associated gamma rays of various energies [26]. The iridium-192 content of each electrode was approximately 250 MBq at delivery.

### Animals

All procedures involving the animals were performed in accordance with the Animals (Scientific Procedures) Act 1986 as amended in 2012 (ASPA) which complies with EU Directive 2010/63/EU, including review and approval by the local Animal Welfare and Ethical Review Body. The animals used were female rats (Wistar-Kyoto (WKY/NHsd), Harlan, UK, supplied specific pathogen free). At the time of exposure the animals were aged between 8 and 12 weeks, weighing between 130 and 180 g. The animals were maintained on a 12 h light/dark cycle within a Scantainer^TM^ ventilated cabinet (Scanbur, Karlslunde, Denmark) in groups of 3 in polycarbonate cages, 1291H Eurostandard Type III H (Tecniplast, Italy), meeting regulatory housing requirements (Code of Practice for the Housing and Care of Animals Bred, Supplied or Used for Scientific Purposes, Home Office (2014), as issued under ASPA). Animals were welfare checked daily and food (Type RMI, Special Diet Services, Witham, Essex, UK) and municipal water were freely available at all times pre- and post-exposure.

Directly following exposure to a radioactive iridium aerosol the whole body iridium-192 content of all animals were measured (see section on radioactive sample counting). The whole body iridium-192 content of live rats was also measured at a number of times post-exposure. Rats to be sacrificed at later times were removed from their restraining tubes and maintained in standard stock cages. The remaining rats were euthanized immediately (i.e. within 15 mins) on completion of the exposure. Animals were euthanized by lethal injection of pentobarbital sodium (450 mg/kg) administered intraperitoneally. To minimise cross contamination during dissection the dead animal was first secured in the supine position on a clean dissection board and the neck, chest and abdominal pelt then damped with a 70% ethanol solution. Dissection equipment was frequently exchanged for clean to avoid the spread of loose radioactive material. The abdomen and thoracic cavity were opened to expose the larynx, major veins were exposed and a blood sample withdrawn using needle and syringe (for a number of animals an alternative approach was used in which a blood sample was taken from the lower left hand heart ventricle), the trachea was tied off close to the larynx, and then the peritoneal cavity opened and the liver, kidneys and spleen removed. The lung, GIT, head, head pelt and body pelt were also removed. For a number of animals brains were also removed. The organs, tissues and remaining carcass were weighed, placed in sample containers, and refrigerated prior to radioactive counting. For a number of animals bronchoalveolar lavage was undertaken prior to the removal of the lung. A cannula was gently inserted and tied in place in the trachea and chilled phosphate buffered saline (PBS) was administered (10 × 10 mL) and aspirated slowly through the needle hub. Each BALF sample was collected separately, the iridium-192 content was measured and then the sample was centrifuged at 500 xg for 5 min at 4 °C to separate the cells from the supernatant. For a number of animals the iridium-192 content of the cell pellet and supernatant samples were measured. For a number of animals BALF samples were then subject to further cytotoxicological analysis (see below). A number of BALF cell samples were fixed in 10% neutral-buffered formalin for later imaging. A number of animals from the main study were placed in metabolism cages at various times post-exposure and faeces and urine collected and refrigerated prior to radioactive counting.

Animals exposed to non-radioactive iridium aerosols were sacrificed using the same procedure as above. Bronchoalveolar lavage was performed on half of the animals using the protocol indicated above. For the other animals the lung was perfused slowly with approximately 200 mL of ice cold saline until the lung turned white. Following vascular perfusion, the lungs were removed en bloc and inflated and fixed with 2% gluteraldehyde in buffer (0.1 M sodium cacodylate) at pH7.3, via the tracheal cannula, at a pressure of 30 cm of water. For electron microscopy the left lung lobe was rinsed briefly in buffer (0.1 M with respect to sodium cacodylate) at pH7.3 and 1 mm thick slices were cut from the central region prior to processing for TEM imaging. Liver, kidneys, brain and BALF cells were fixed by infusion with 10% neutral-buffered formalin. For laser ablation ICP-MS and synchrotron X-ray spectroscopy analysis three consecutive 5 μm sections were cut from the centre of each organ (lung (diaphragmatic lobe), liver, brain and kidney).

### Radioactive sample counting

The iridium-192 content of each sample was measured using either a 1480 Wizard^TM^ 3” Automatic Gamma Counter using MultiCalc software (Perkin Elmer, MA, USA) for small samples (e.g. whole kidney, spleen, brain, blood and daily excreta) or a Scintiflex 178 YP 254 NaI “Well Counter” (Nuclear & Silica Products Ltd, High Wycombe, UK) using Ortec Maestro software (ORTEC, TN, USA) for larger samples (e.g. whole carcass, pelt). The whole body content of live rats was determined using the Well Counter. Animals were placed in exposure tubes and inserted horizontally into the well counter for up to 30 min. The iridium-192 content (Bq) of a sample was determined by subtracting the background count rate from the sample count rate for the detector and dividing by the appropriate (i.e. counter and sample type) detection efficiency. Sample activities were normalised to the day of the exposure by correcting for radioactive decay. Minimum detectable activity (MDA) levels were calculated for each sample [27] and only values above the MDA reported.

### BALF cytotoxicity analysis

Cells from all the BALF were collected in 1 mL saline and placed on ice. Cells were counted using a haemocytometer under a microscope. Total BALF cell numbers were calculated and around 1 × 10^5^ cells were precipitated onto cytospin slides for cell population counting. Staining was performed with Diff-Quick^TM^, following the manufacturer’s instructions. For each cytospin slide, at least 300 cells were counted to identify macrophages, neutrophils, and lymphocytes according to standard morphology under a microscope with x400 magnification.

Total protein, lactate dehydrogenase (LDH) and alkaline phosphatase (ALP) assays were used to assess general cytotoxicity using the supernatant of the first bronchoalveolar lavage wash (samples were stored at −80 °C before analysis). Assays were performed in 96-well plates. For LDH assay (Promega), 50 μl of BALF was added to 50 μl of reconstituted substrate solution and incubated for 30 mins at room temperature in the dark. Then 50 μl of stop solution was added to each well and the absorbance (Ab) was measured at 492 nm. Total protein was measured using Bio-Rad protein assay kit and ALP was measured using Abcam alkaline phosphatase assay kit.

### Tissue sample imaging

Images of lung sections and BALF cells from animals exposed to the non-radioactive 10 nm and 75 nm aerosols were produced using a Zeiss 902A electron microscope. Following this initial analysis, further images of some of the particles found in the BALF cells were produced using a JEOL 2010 analytical transmission electron microscope with a resolution of 0.19 nm and electron probe size down to 0.5 nm. This instrument was equipped with an Oxford Instruments LZ5 windowless energy dispersive X-ray spectrometer (EDS) to enable elemental analysis.

Laser ablation inductively coupled plasma mass spectrometry (LA-ICP-MS) analyses of lung, liver, kidney and brain sections and BALF cells mounted on microscope slides were undertaken using a New Wave Research NWR213 laser ablation system (Electro Scientific Industries, Portland, Oregon, USA) linked to a iCAP Q ICP-MS (Thermo Fisher Scientific, Hemel Hempstead, UK). Unless otherwise stated, laser ablation conditions comprised 100 μm diameter spot size, fluence 7 J/cm2, 400 μms^−1^ scan speed and a repetition rate of 20Hz. The cell gas was helium which was run at a flow of 0.9 mLmin^−1^. Helium was also used as the collision gas in KED mode. Samples were introduced into the ICP-MS via a micro flow nebuliser. The ICP-MS was run in KED mode and the isotopes monitored were carbon-13, copper-63, zinc-66 and iridium-193. Dwell time for each isotope was 0.05 s. Scan log files were generated to allow reconstruction of the data into an image. Image generation was achieved using Iolite v3 [28] within Igor Pro 6.36 (Wavemetrics Inc. Oregon, USA). Basically, the scan log file was aligned with the ICP-MS data exported from Qtegra as an excel.csv file, to identify data of interest. Baseline values were then subtracted using the data reduction scheme using carbon-13 as the index channel.

Synchrotron micro focus x-ray fluorescence (μ-XRF) was used to produce elemental maps of lung sections and BALF cells. For identified areas of high iridium concentration micro focus X-ray near-edge absorption spectroscopy (μ-XANES) was used to explore iridium speciation via comparison with spectra produced for a range of reference samples, including iridium wire, iridium nanoparticles sampled from the aerosol onto a filter, iridium oxide (Sigma Aldrich, Gillingham, UK), iridium chloride (Fisher Scientific, Loughborough, UK), and iridium dodecacarbonyl (Sigma Aldrich, Gillingham, UK). This was undertaken using the microfocus spectroscopy beamline, I18 [29], at the Diamond Light Source. The μ-XRF mapping of iridium and other elements was undertaken using a beam focus of circa 4 × 4 μm. The iridium L(III)-edge spectra was used for the μ-XANES analysis with a step size of 5 eV for the majority of the spectra reducing to 0.5 eV where more detailed resolution was required.

## Additional files

Additional file 1: Supplementary Material: Tables S1-S4 and Figures S1-S14. (PDF 2293 kb)

Additional file 2: Ir-192 content of organs and tissues. (XLSX 22 kb)

## Abbreviations

μ-XANES: Synchrotron microfocus x-ray near-edge absorption spectroscopy
μ-XRF: Synchrotron microfocus x-ray flourescence
ALP: Alkaline phosphatase
BALF: Bronchoalveolar lavage fluid
CMD: Count median diameter
DMA: Differential mobility analyser
EDS: Energy dispersive x-ray spectrometer
GIT: Gastrointestinal tract
GSD: Geometric standard deviation
H&E: Haematoxylin and eosin
LA-ICP-MS: Laser ablation inductively coupled mass spectrometry
LDH: Lactate dehydrogenase
MDA: Minimum detectable activity
NAS: Nanometer aerosol sampler
POA: Point of administration
SMPS: Scanning mobility particle sizer
TEM: Transmission electron microscopy
