# Supplementary material for: Slow lung clearance and limited translocation of four sizes of inhaled iridium nanoparticles
Source: Part Fibre Toxicol. 2017 Feb 10;14:5. doi: 10.1186/s12989-017-0185-5 (PMC5304551; doi:10.1186/s12989-017-0185-5)
Supplement: Supplementary file 1 — Supplementary Material: Tables S1-S4 and Figures S1-S14. (PDF 2293 kb) [file 12989_2017_185_MOESM1_ESM.pdf]

ADDITIONAL FILE 1

Table S1 Aerosol characteristics for supplementary iridium-192 study

Exposure duration (min)	CMD (nm)	GSD	Particle number concentration (x 10^{13} #/m^3)	Activity concentration (kBq/m^3)	Mass concentration (mg/m^3)
10	16.8 ± 0.1	1.654 ± 0.004	8.28 ± 0.11	599 ± 100	1.00 ± 0.17
20	16.7 ± 0.1	1.652 ± 0.005	8.07 ± 0.10	593 ± 99	0.99 ± 0.17
40	16.6 ± 0.1	1.654 ± 0.006	8.12 ± 0.09	607 ± 102	1.02 ± 0.17
60	16.6 ± 0.1	1.654 ± 0.006	8.17 ± 0.18	579 ± 97	0.97 ± 0.16

Table S2 Aerosol characteristics for non-radioactive iridium aerosol

Nominal particle diameter (nm)	CMD (nm)	GSD	Particle number concentration (x 10^{13} #/m^3)	Mass concentration (mg/m^3)
10	11.8 ± 0.2	1.52 ± 0.01	2.31 ± 0.19	0.24 ± 0.01
75	81.6 ± 1.6	1.65 ± 0.01	1.14 ± 0.02	8.82 ± 1.72

Table S3 Iridium-192 content of BALF as percentage of total lung content

Nominal particle diameter (nm)	Post-exposure time (d)	BALF (Bq)/ Lung (Bq)	Supernatant (Bq)/ BALF (Bq)	Cellular fraction (Bq)/ BALF (Bq)
10	0	37 ± 7%	74 ± 15%	29 ± 6%
10	3	30 ± 5%	20 ± 4%	89 ± 19%
10	7	28 ± 6%	18 ± 7%	91 ± 27%
10	30	36 ± 16%	-	-
10	90	42 ± 10%	-	-
35	3	36 ± 7%	17 ± 5%	83 ± 22%
35	28	34 ± 5%	16 ± 4%	84 ± 18%

Table S4 Estimated pulmonary deposited doses

Nominal particle diameter (nm)	Exposure duration (mins)	Deposited mass* (µg/animal)
Main study – clearance and translocation		
10	120	1.1
15	75	5.0
35	180	25.6
75	60	8.9
Supplementary study A – clearance		
15	10	0.5
15	20	0.9
15	40	1.9
15	60	2.8
Supplementary study B – extra imaging samples		
10	120	1.5
75	60	11.3

*Deposited mass = aerosol mass concentration x exposure duration x breathing rate x deposition efficiency. The breathing rate assumed was 0.16 l/min, on the basis of our measurements on similar animals in the same exposure system (i.e. breathing frequency 127 breaths/min and tidal volume 1.3 ml [1]). Deposition efficiencies were determined using MPPD v2.11 [2], using rat respiratory parameters considered appropriate for the rat strain and size used, including the aforementioned breathing frequency and tidal volume, an FRC value of 3.7 ml, Filho et al [3], and a URT volume of 0.29 ml, derived by body mass scaling according to the method of Ménache et al [4].

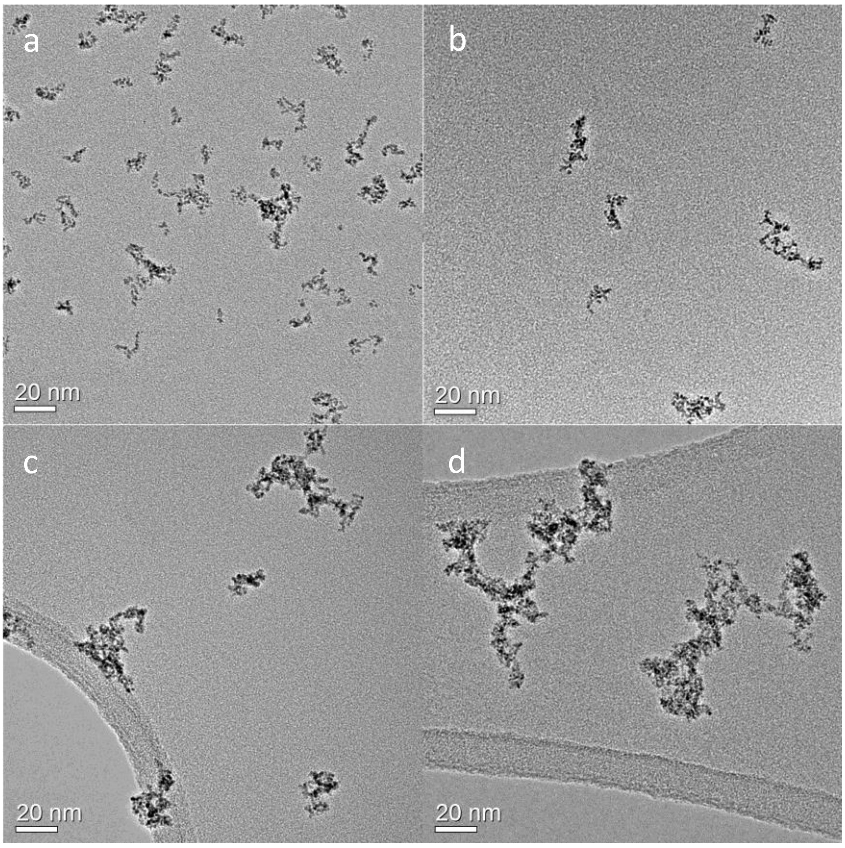

Figure S1. Representative transmission electron micrographs of iridium-192 aerosol particles, nominal size (a) 10 nm, (b) 15 nm, (c) 35 nm and (d) 75 nm.

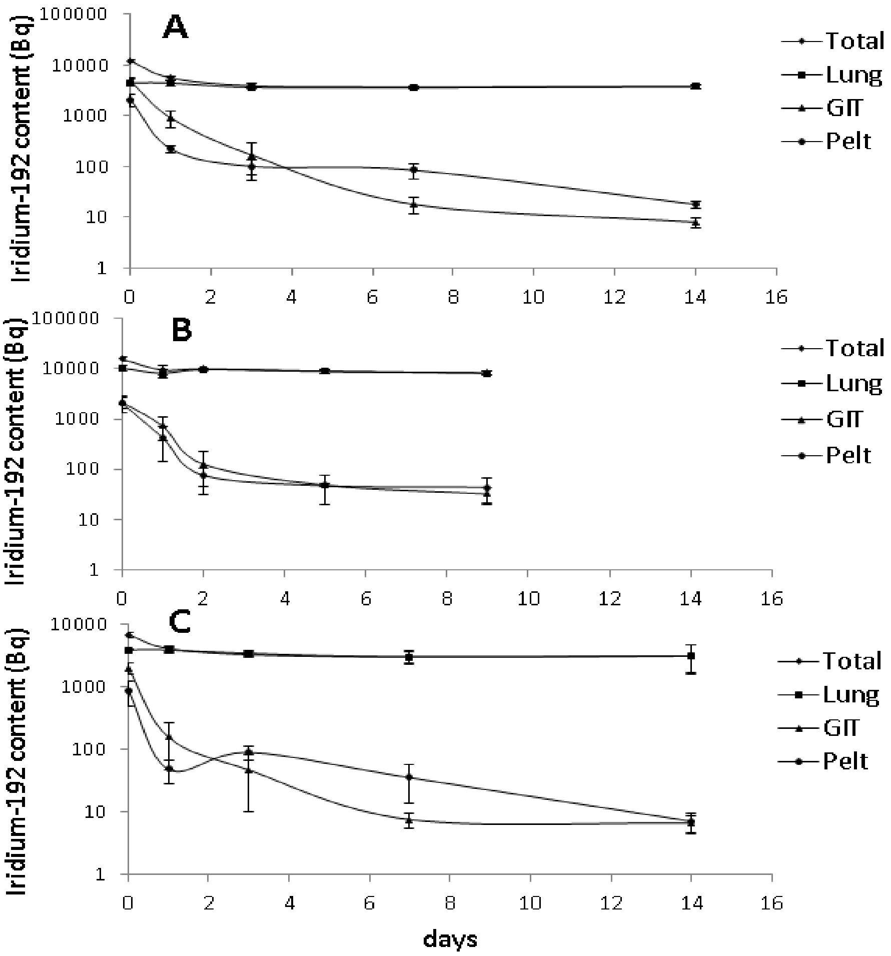

Figure S2. Average iridium-192 content of the whole-body, lung, gastrointestinal tract (GIT), and pelt during the first two weeks post-exposure to iridium-192 aerosols: (A) 10 nm, (B) 15 nm, and (C) 35 nm (n ≥ 4). Note that the results for the 75 nm aerosol have not been presented graphically as only two time points are available for this study, 0 and 30 days post-exposure. At day 0 the iridium-192 content of the lung, GI tract, and pelt for this aerosol were, respectively, 1360 ± 125 Bq, 527 ± 85 Bq and 576 ± 109 Bq.

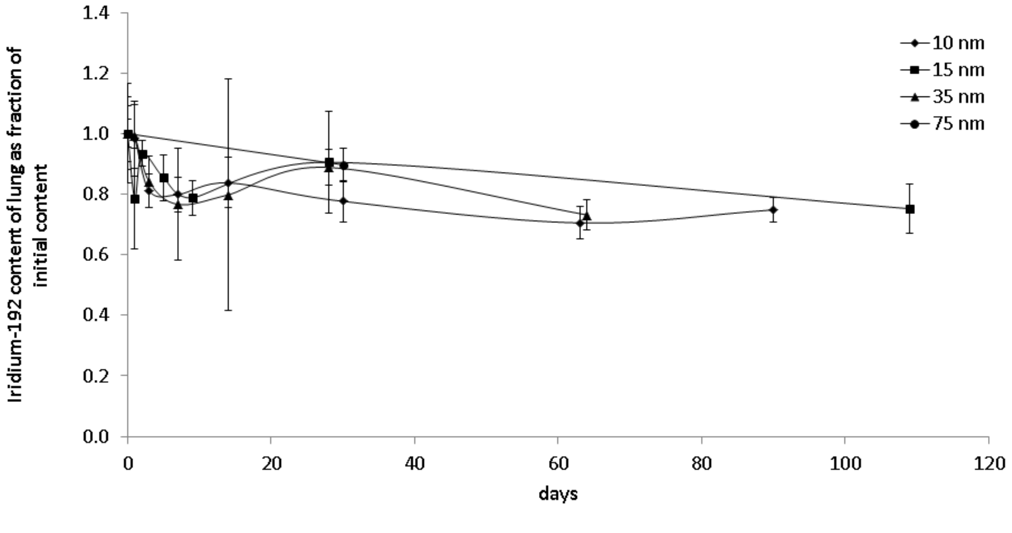

Figure S3. Average iridium-192 content of lung as a fraction of the lung content at day 0 following exposure to iridium-192 aerosols of 10 nm, 15 nm, 35 nm, and 75 nm (n ≥ 4).

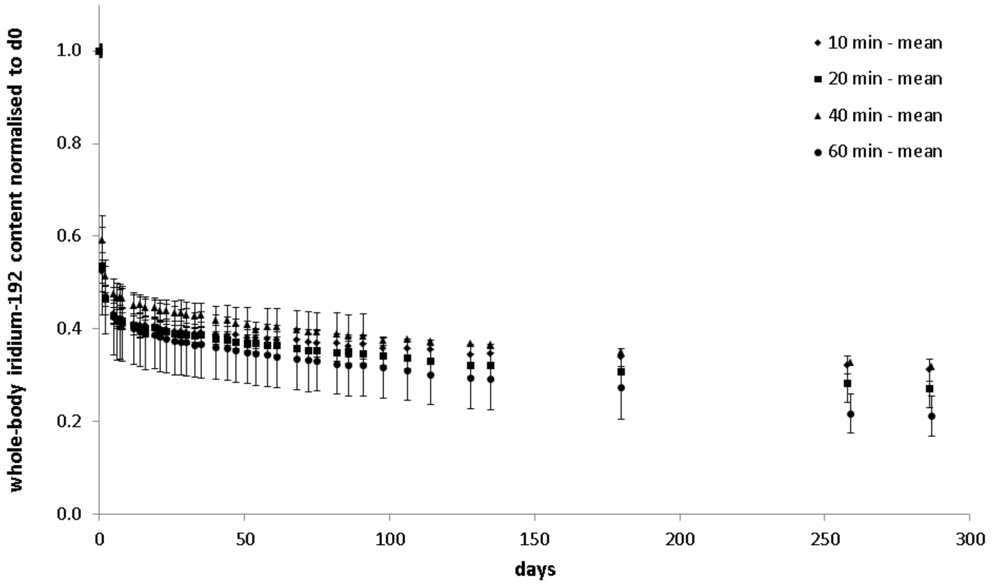

Figure S4. Average whole body iridium-192 content normalised to day 0 for animals exposed to 15 nm iridium-192 aerosol for 10 min, 20 min, 40 min, and 60 min (n ≥ 4).

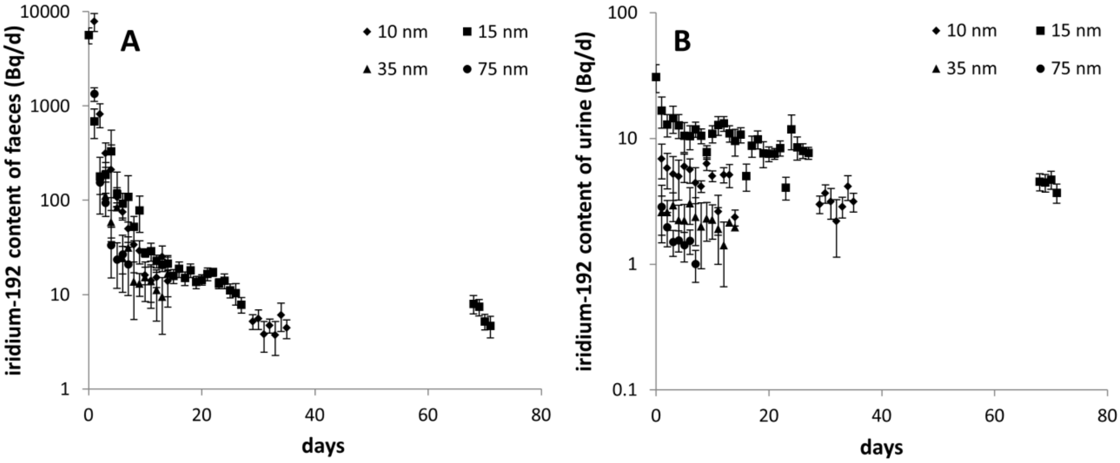

Figure S5. Average iridium-192 content of (A) faeces and (B) urine following exposure to iridium-192 aerosols of 10 nm, 15 nm, 35 nm, and 75 nm (n ≥ 4).

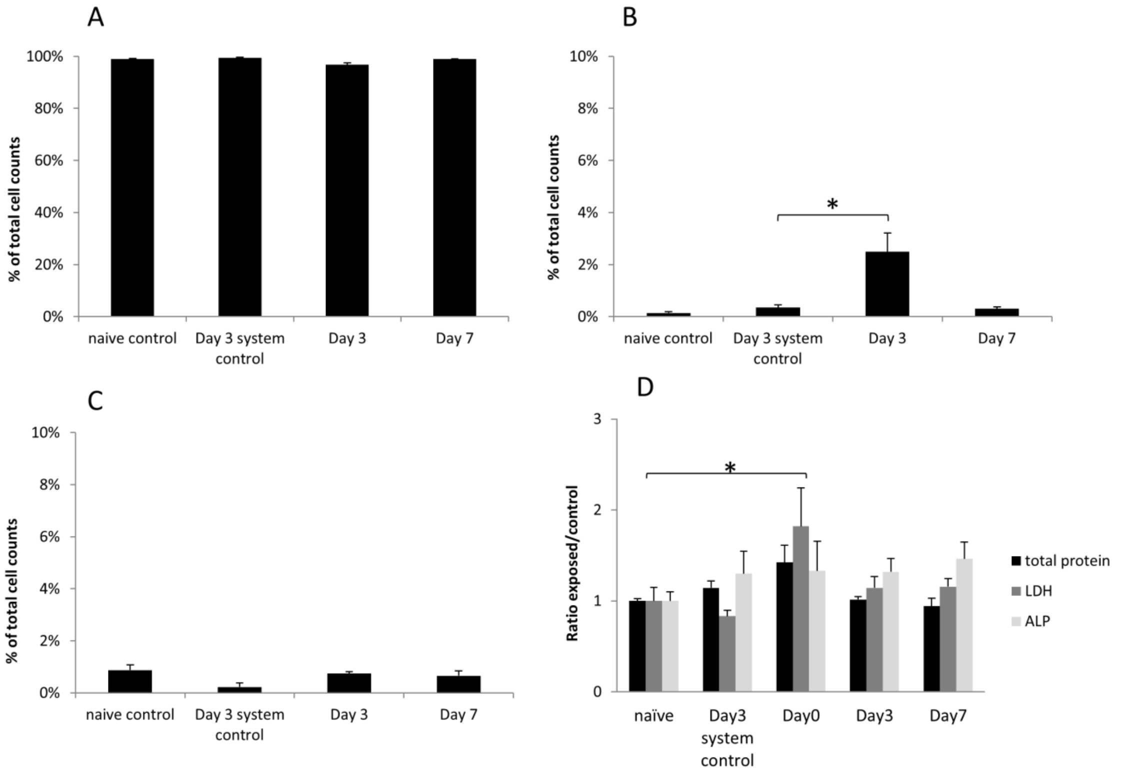

Figure S6. Measures of toxicity using bronchoalveolar fluid following exposure to 10 nm iridium-192 aerosol (n=4): (A) macrophages (B) PMN and (C) lymphocytes as percentage of total cell numbers - there were no significant changes to total cell numbers ($4.1 \pm 0.4 \times 10^6$) or numbers of macrophages, or lymphocytes; (D) total protein, lactate dehydrogenase, and alkaline phosphatase (* $p < 0.05$). Results indicate a low level of response resolving by day 7.

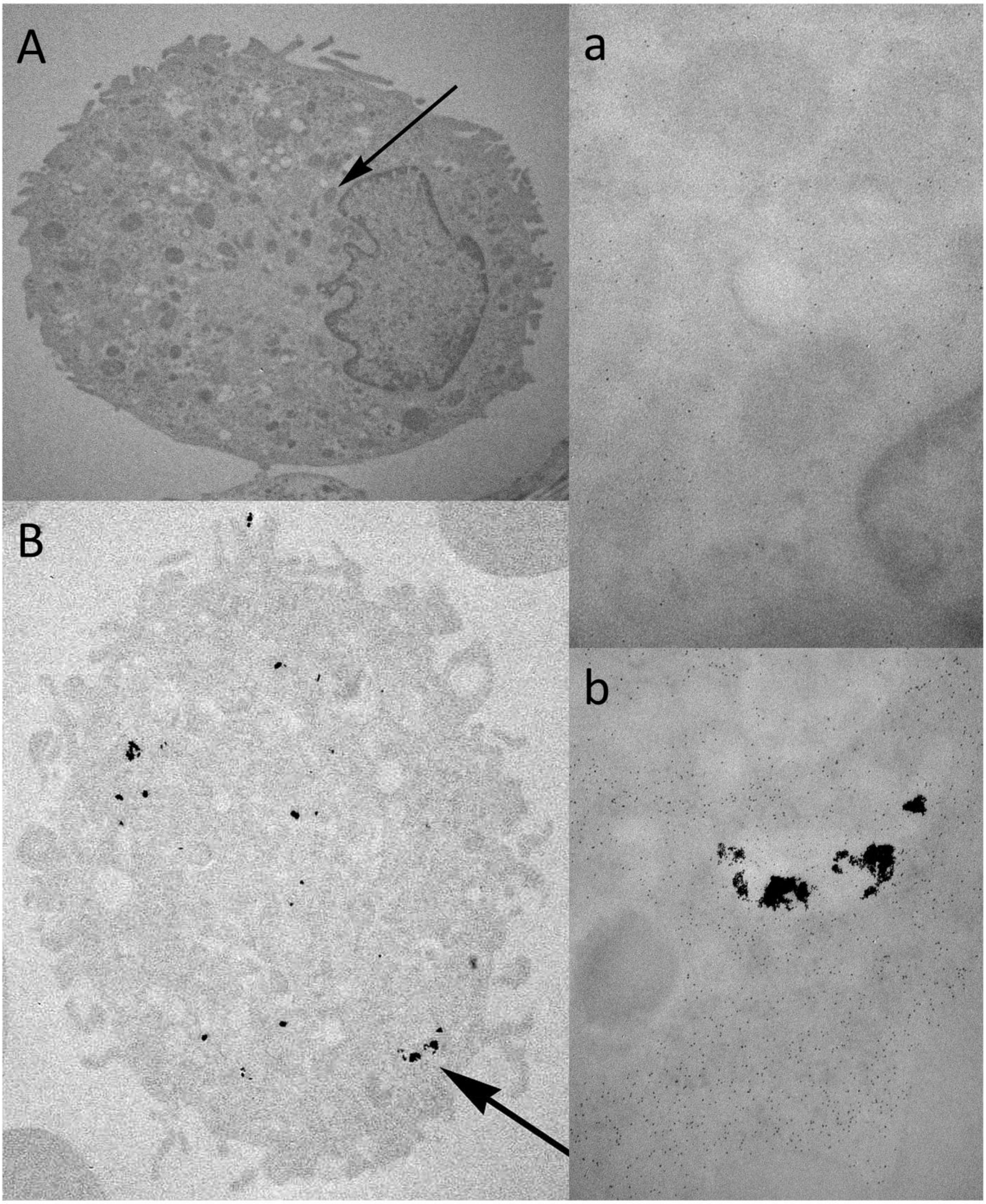
A
a
B
b

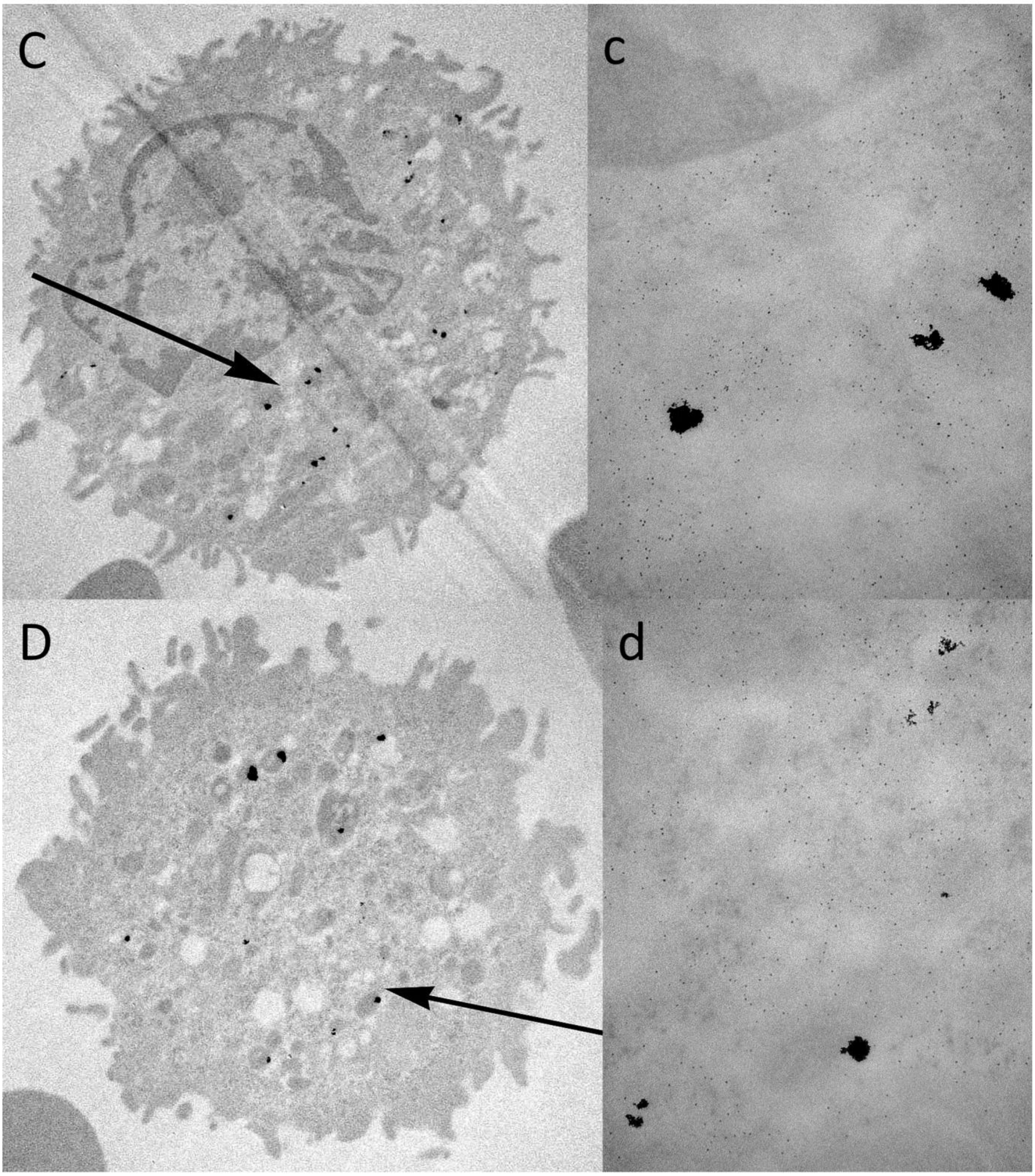

Figure S7. Transmission electron micrographs (x20,000) of cells from BALF cell samples from (A) control animal, (B) animal exposed to 75 nm iridium aerosol at 1 day post-exposure, and (C, D) animal exposed to 75 nm iridium aerosol at 5 days post-exposure. Higher magnification (x100,000) images of the parts of the cells indicated by arrows are identified by the equivalent lower case letters (a, b, c and d, respectively). Images for the exposed animals show the presence of dense particulates.

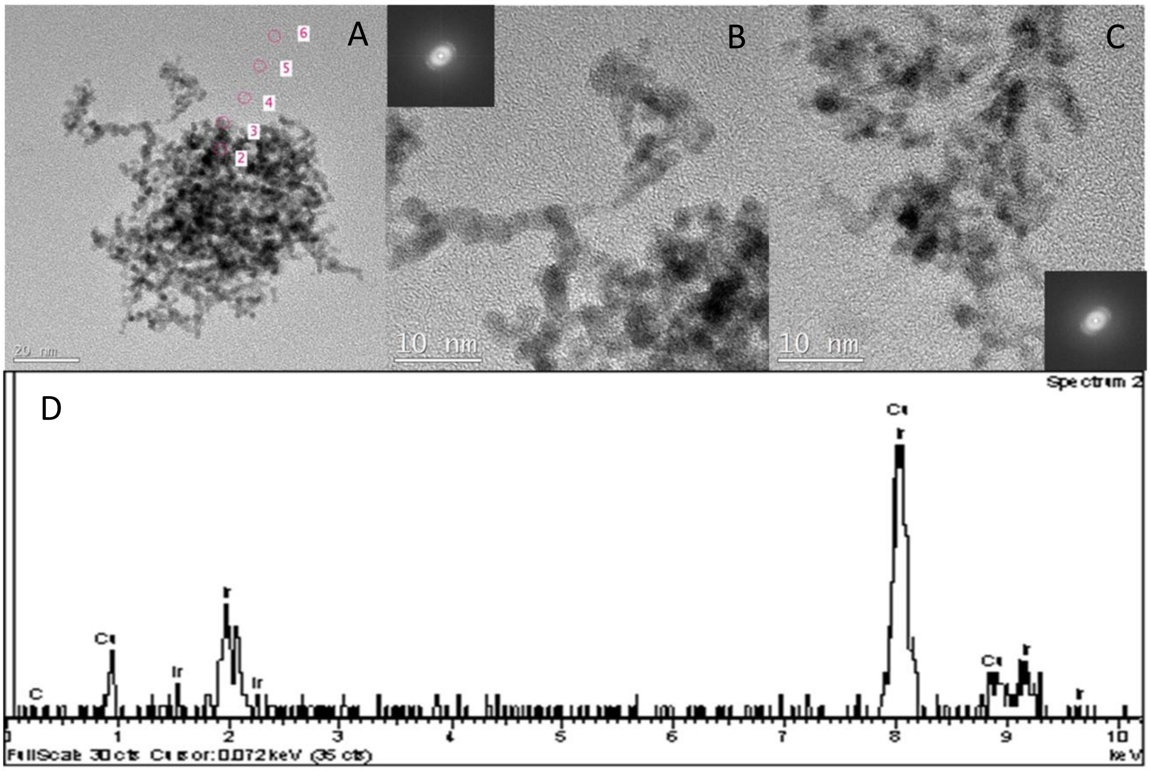

Figure S8. Transmission electron micrographs of (A) iridium particle identified in BALF cell 1 day post-exposure to 75 nm iridium aerosol indicating positions at which energy-dispersive X-ray spectroscopy (EDX) analysis was undertaken, (B) an additional image of part of the particle with X-ray diffraction pattern indicating crystalline structure, and (C) iridium particle identified in BALF cell at 30 days post-exposure to 75 nm aerosol, with associated x-ray diffraction pattern indicating crystalline structure. EDX spectrum for position 2 in image A indicating the clear presence of iridium (D). The spectrum at position 3 was similar, but only very low levels of iridium were found at positions 4, 5, and 6. Spectra for other particles were similar.

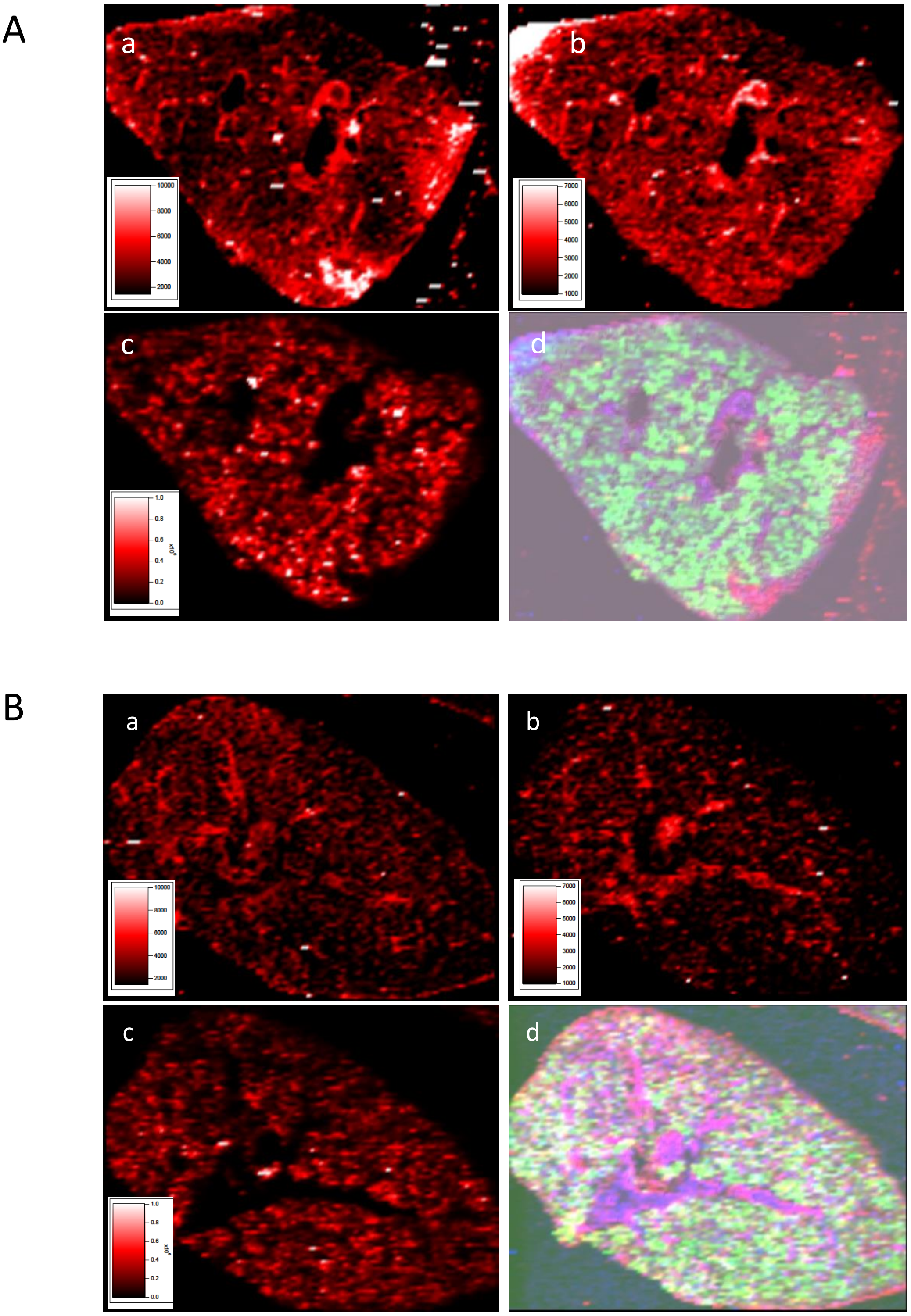

Figure S9. Laser ablation inductively coupled plasma mass spectrometry (LA-ICP-MS) elemental maps of lung tissue collected at 1 day (A) and 30 days (B) post-exposure to 75 nm aerosol, showing the distribution of copper (a), zinc (b), iridium (c) and an overlay of all three plots (copper, red; zinc, blue; iridium, green) (d). The spatial pattern of the iridium was similar at all times (1, 5 and 30 days post-exposure) and for both particle sizes (10 nm and 75 nm). The copper and zinc distribution maps are included to enhance visualisation of the localisation of the iridium; results from control animals (not shown) indicate no iridium is present and the same overall pattern of distribution of copper and zinc.

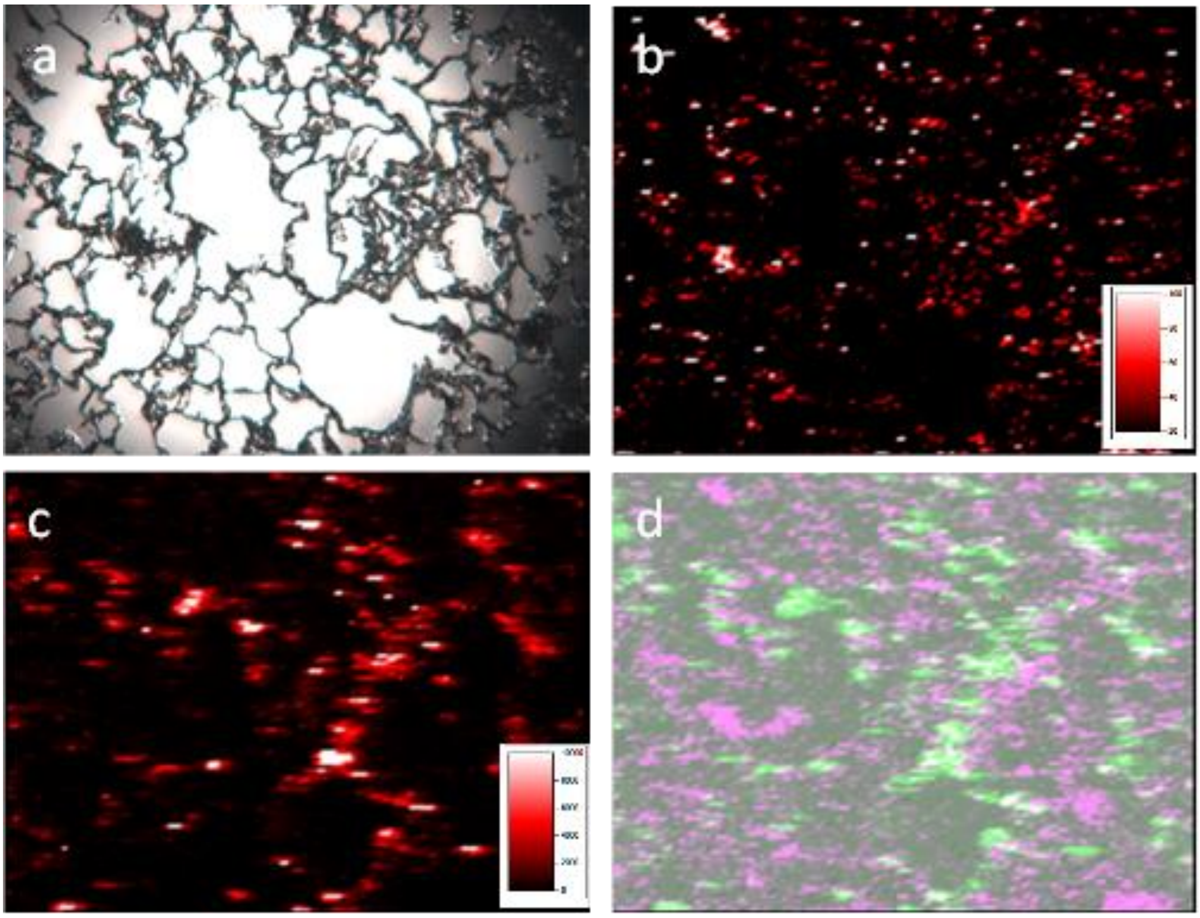

Figure S10. Laser ablation inductively coupled plasma mass spectrometry (LA-ICP-MS) elemental maps and associated light microscopy image of lung tissue section from rat at 1 day post-exposure to 75 nm iridium aerosol. Light microscopy image showing the bronchoalveolar region prior to ablation (a), distribution of zinc (b), distribution of iridium (c), and an overlay of zinc and iridium plots (zinc, magenta; iridium, green) (d). Changes to the LA method described in Methods were; spot size, 5 µm, laser strength 50% and scan speed 25 µm/s. The zinc distribution map is included to enhance visualisation of the localisation of the iridium; results from control animals (not shown) indicate no iridium is present and the same overall pattern of zinc distribution.

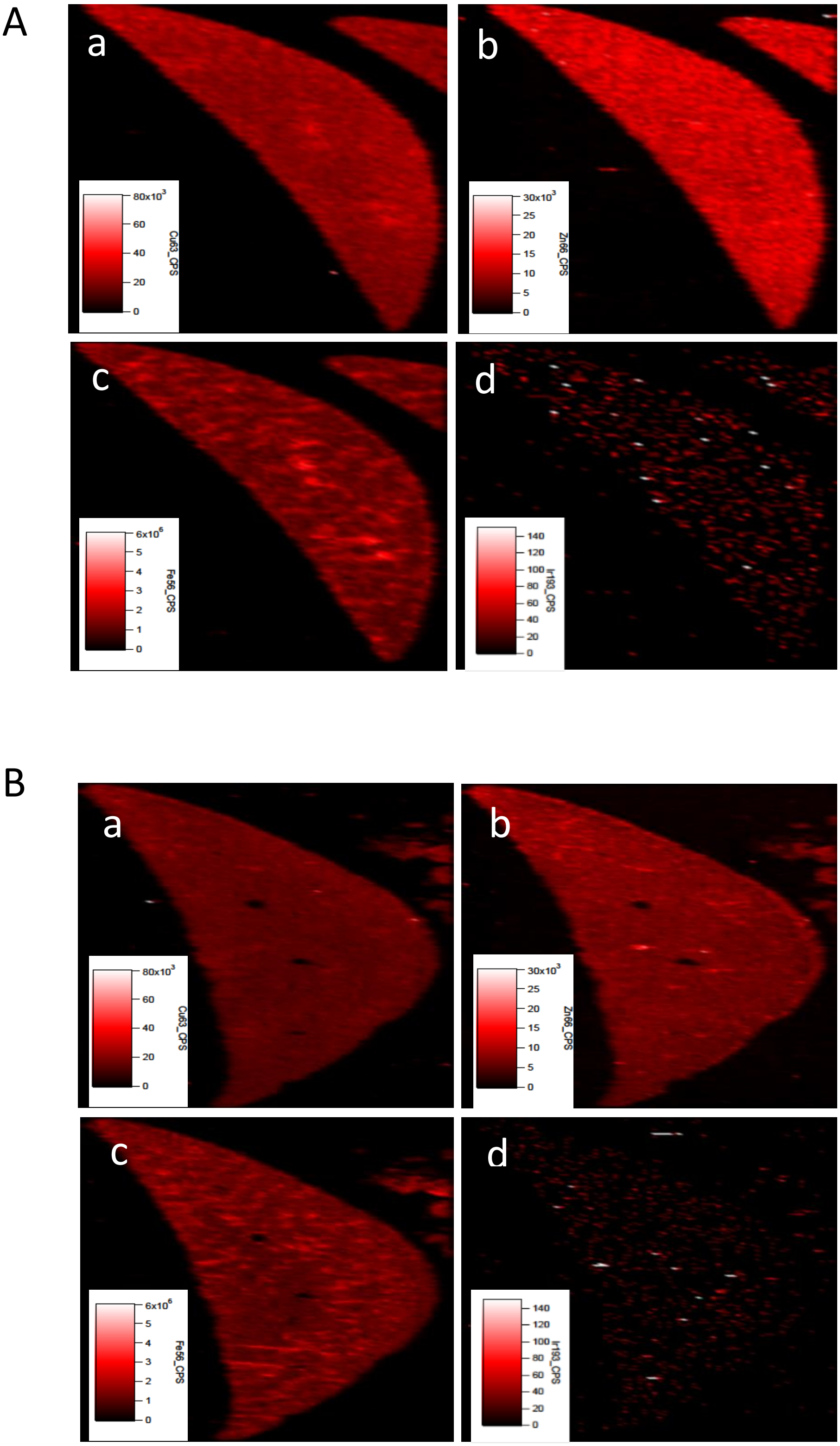

Figure S11. Laser ablation inductively coupled plasma mass spectrometry (LA-ICP-MS) elemental maps of liver sections at 30 days post-exposure to (A) 10 nm and (B) 75 nm iridium aerosols showing the distribution of copper (a), zinc (b), iron (c) and iridium (d). The copper, zinc and iron distribution maps are included to enhance visualisation of the localisation of the iridium; results from control animals (not shown) indicate no iridium is present and the same overall pattern of distribution of copper, zinc and iron.

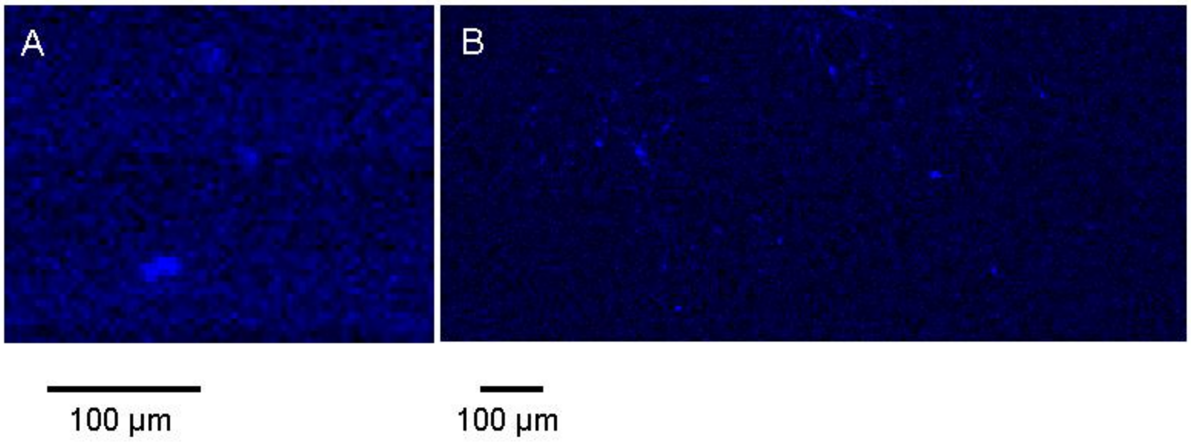

Figure S12. Elemental maps (µ-XRF) of iridium in (A) BALF cell pellet from animals exposed to 10 nm aerosol at 3 days post-exposure, and (B) lung tissue sample from animals exposed to 10 nm aerosol at 1 day post-exposure, obtained using the I18 beamline at the Diamond Light Source (pixel size – 4 µm x 4 µm).

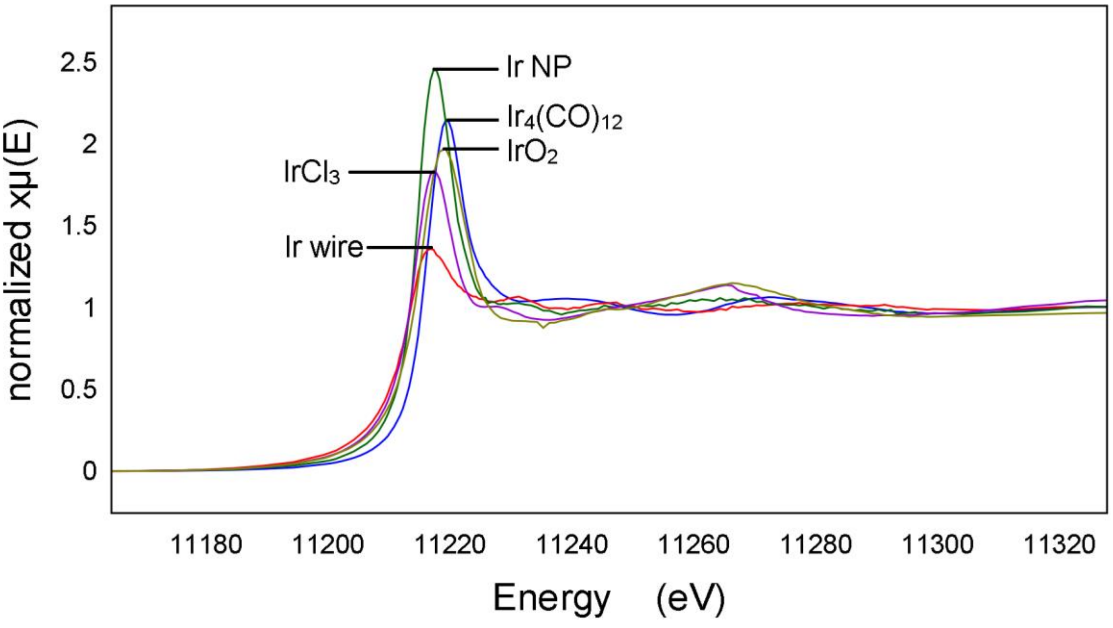

Figure S13. Iridium (L(III)-edge) µ-XANES spectra for various iridium samples. The samples were of different forms: wire (Ir), aerosol nanoparticles on filter (Ir NP), and compressed powder samples (IrO_2, $IrCl_3$, and $Ir_4(CO)_{12}$).

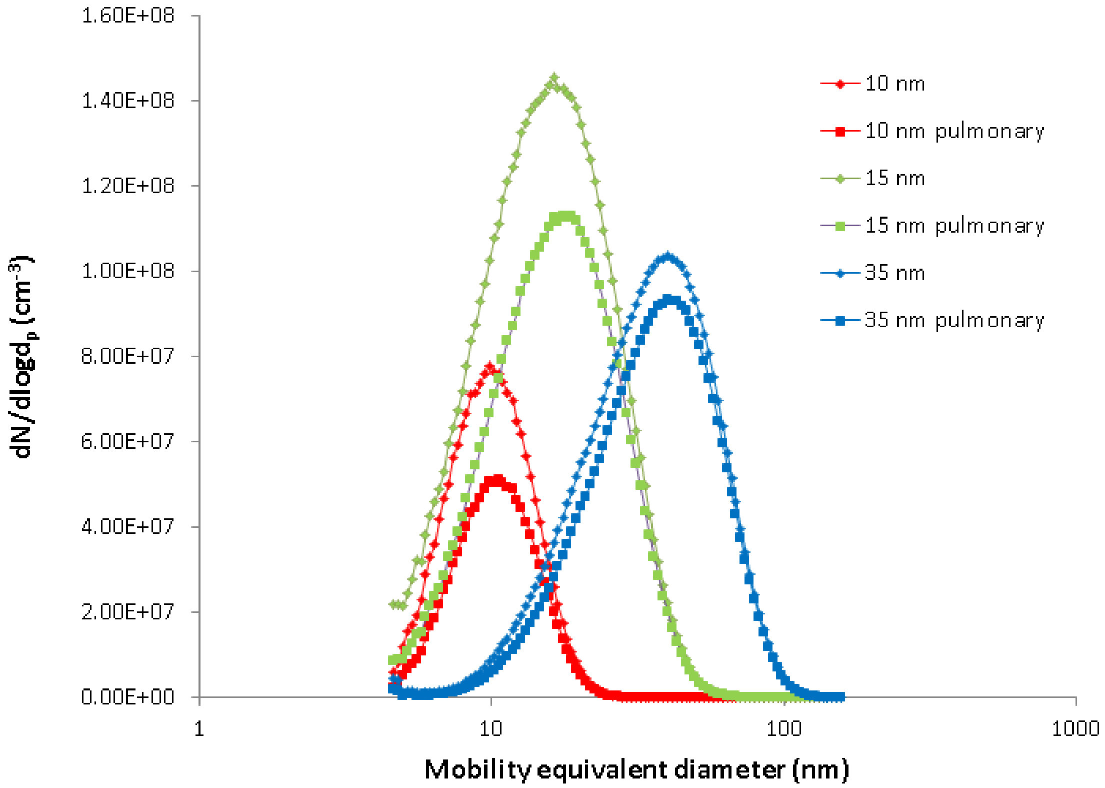

Figure S14. Average mobility equivalent size distribution of iridium-192 aerosols as delivered to the animal and reaching the pulmonary region. The latter were derived by multiplying the former by (1 – DN_d), where DN_d is the deposition efficiency in the nose for particle size d. Values of DN_d were determined using MPPD v2.11 [2]. The results indicate a reduction in the number of the smaller particles reaching the lung (i.e. approximately 45% of particles < 6 nm reach the lung and approximately 60% of particles < 10 nm reach the lung). The overall impact was to reduce the number of particles reaching the lung to 87%, 74% and 64% for the 35 nm, 15 nm and 10 nm aerosols, respectively. The effects on the CMD were minor with an increase of < 1 nm in each case.

References

1. Buckley A, Hodgson A, Warren J, Guo C, Smith, R. Size dependent deposition of inhaled nanoparticles in the rat respiratory tract using a new nose-only exposure system. Aerosol Sci. & Technol. 2016;50(1):1-10.

2. Asgharian B, Price O, Miller F, Subramaniam R, Cassee FR, Freijer J, van Bree L, de Winter-Sorkina R. MPPD - Multiple-Path Dosimetry Model v2.11 (2009). Applied Research Associates (ARA), The Hamner Institutes for Health Sciences, the National Institute of Public Health and the Environment (RIVM), the Netherlands, and the Ministry of Housing, Spatial Planning and the Environment, the Netherlands.

3. Filho WJ, Fontinele RG, de Souza, RR. Reference Database of Lung Volumes and Capacities in Wistar Rats from 2 to 24 Months. Current Aging Science 2014;7(3):220-228.

4. Ménache MG, Hanna LM, Gross EA, Lou SR, Zinreich SJ, Leopold DA, Jarabek AM, Miller FJ. Upper respiratory tract surface areas and volumes of laboratory animals and humans: considerations for dosimetry models. J. Toxicol. Environ. Health: Current Issues, 1997;50(5):475-506.
